# Supplementary figures and images for: SUCLG1 restricts POLRMT succinylation to enhance mitochondrial biogenesis and leukemia progression (part 1 of 2)
Source: EMBO J. 2024 Apr 22;43(12):2337–67. doi: 10.1038/s44318-024-00101-9 (PMC11183053; doi:10.1038/s44318-024-00101-9)

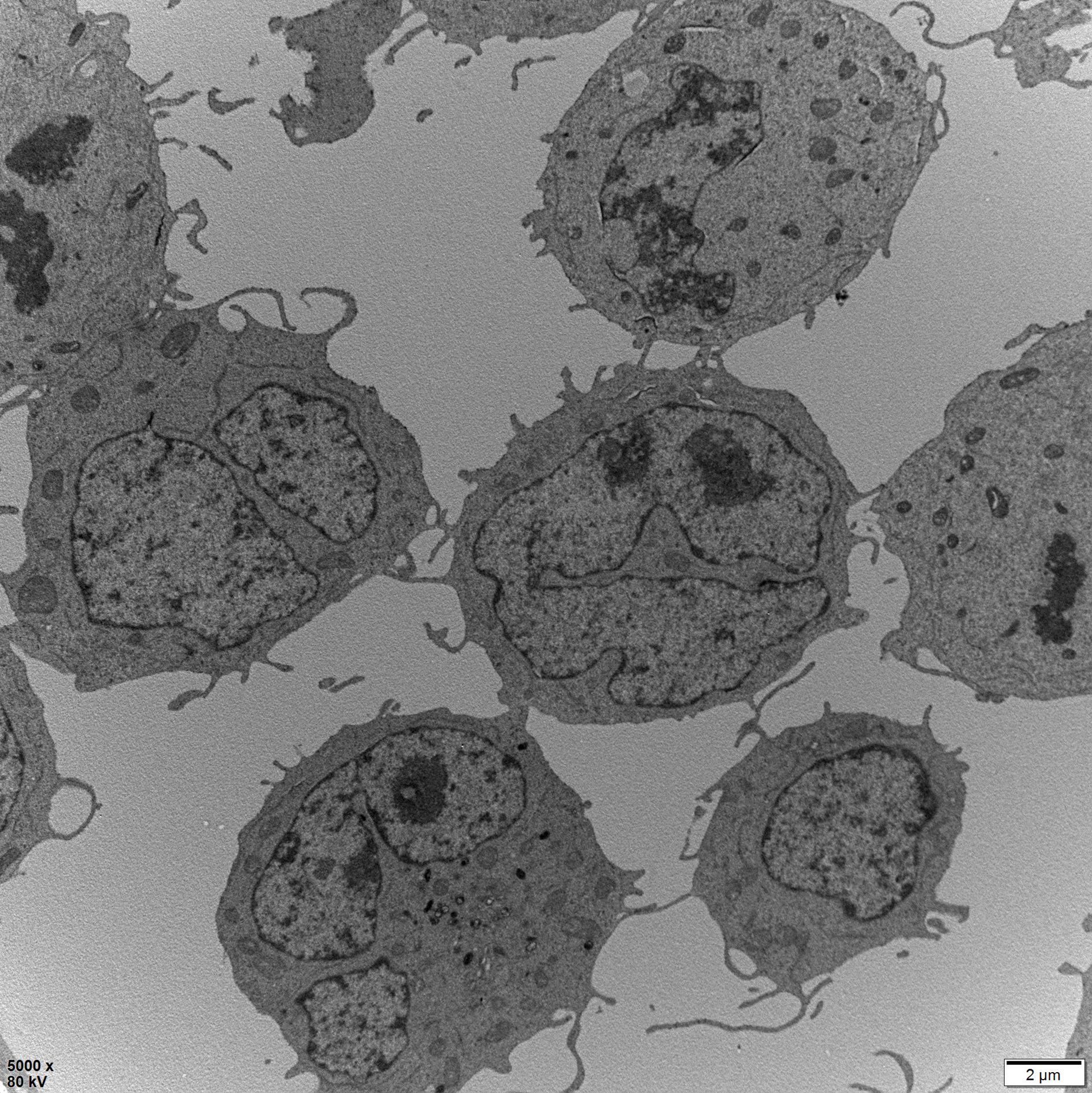

Supplement: Supplementary file 2 — Source data Fig. 1 [file 44318_2024_101_MOESM2_ESM.zip › Figure 1/1G/1G MV411 shSUCLG1.tif]

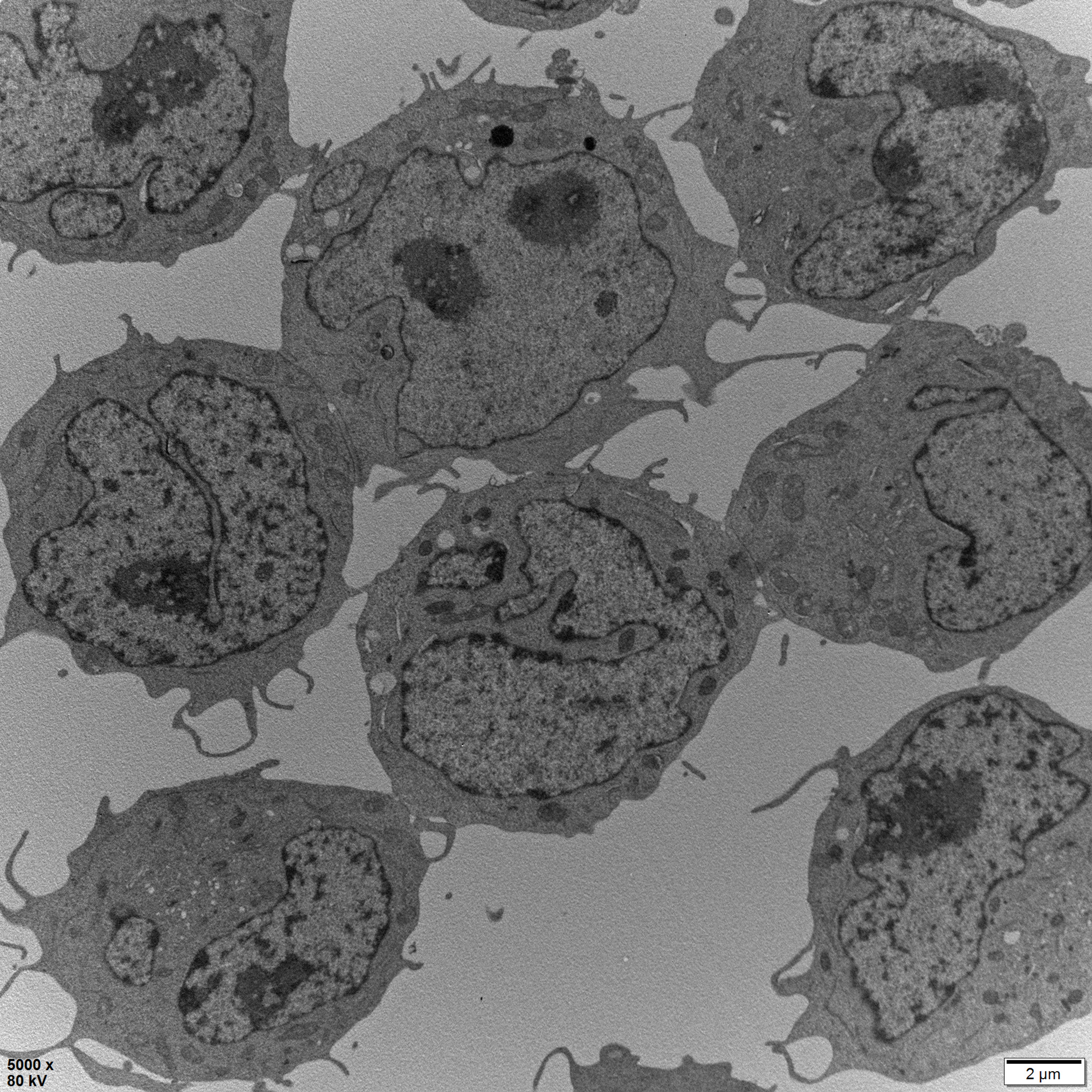

Supplement: Supplementary file 2 — Source data Fig. 1 [file 44318_2024_101_MOESM2_ESM.zip › Figure 1/1G/1G MV411 scr.tif]

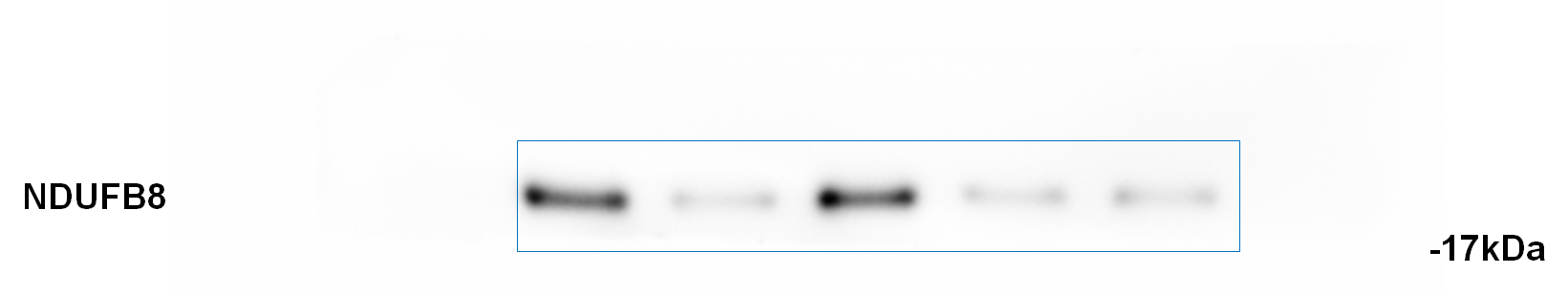

Supplement: Supplementary file 2 — Source data Fig. 1 [file 44318_2024_101_MOESM2_ESM.zip › Figure 1/1I/western NDUFB8.tif]

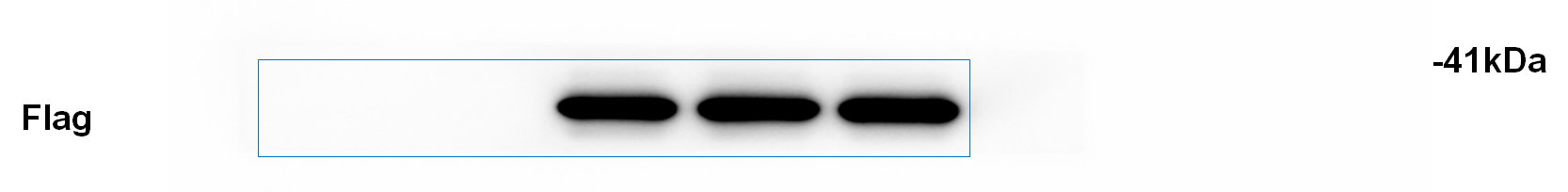

Supplement: Supplementary file 2 — Source data Fig. 1 [file 44318_2024_101_MOESM2_ESM.zip › Figure 1/1I/western Flag.tif]

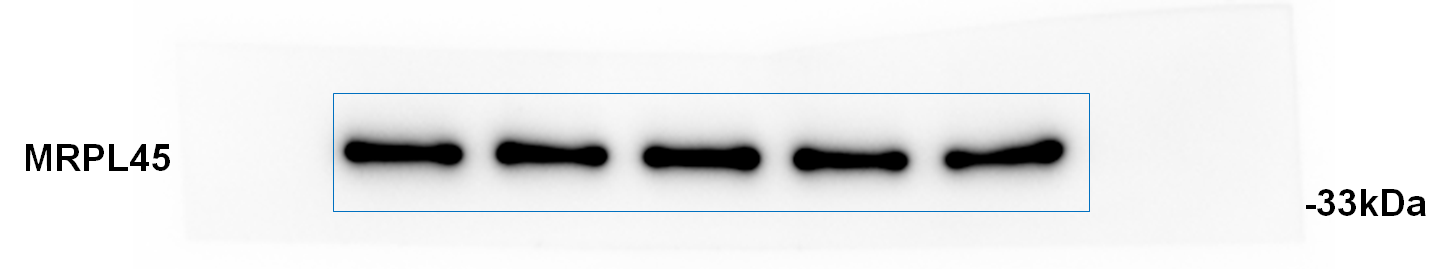

Supplement: Supplementary file 2 — Source data Fig. 1 [file 44318_2024_101_MOESM2_ESM.zip › Figure 1/1I/western MRPL45.tif]

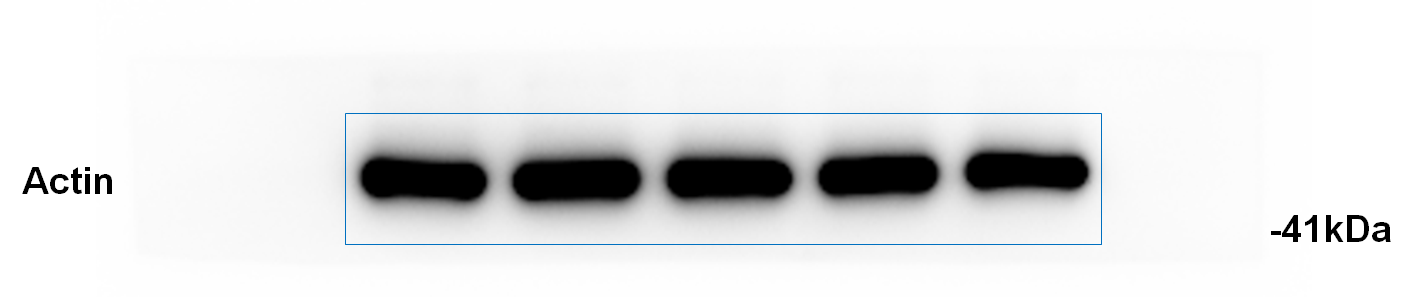

Supplement: Supplementary file 2 — Source data Fig. 1 [file 44318_2024_101_MOESM2_ESM.zip › Figure 1/1I/western Actin.tif]

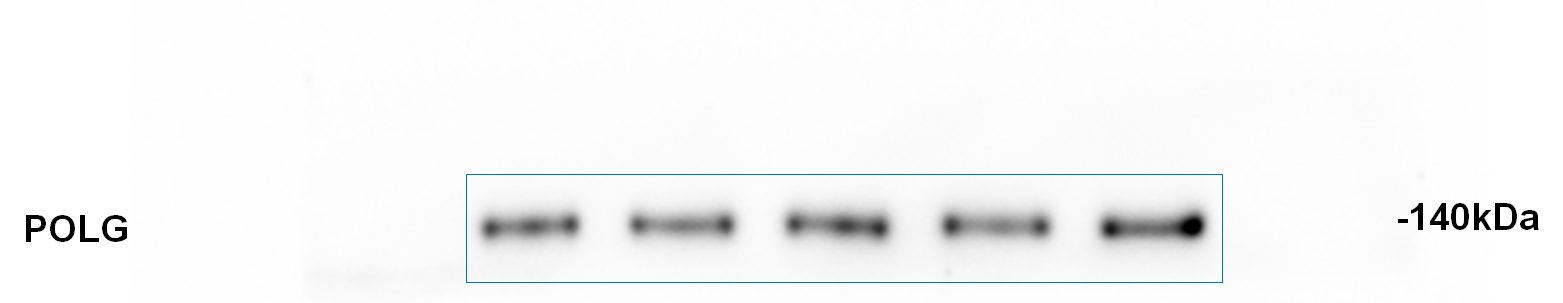

Supplement: Supplementary file 2 — Source data Fig. 1 [file 44318_2024_101_MOESM2_ESM.zip › Figure 1/1I/western POLG.tif]

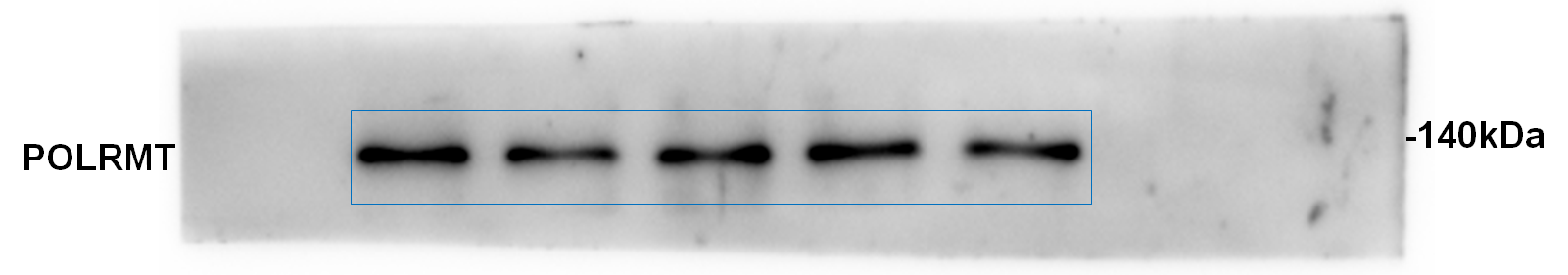

Supplement: Supplementary file 2 — Source data Fig. 1 [file 44318_2024_101_MOESM2_ESM.zip › Figure 1/1I/western POLRMT.tif]

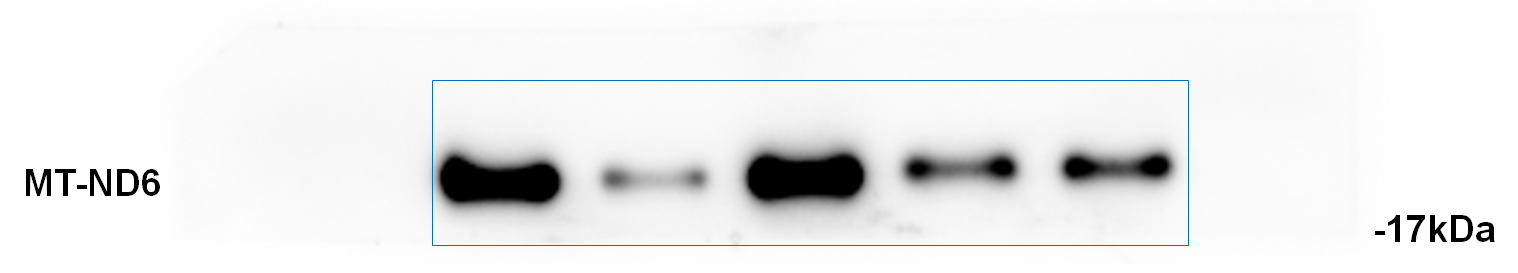

Supplement: Supplementary file 2 — Source data Fig. 1 [file 44318_2024_101_MOESM2_ESM.zip › Figure 1/1I/western MT-ND6.tif]

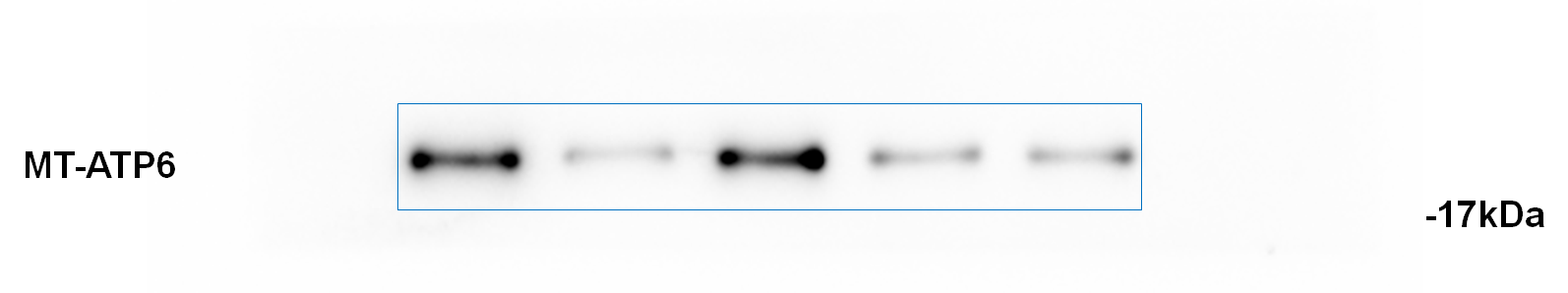

Supplement: Supplementary file 2 — Source data Fig. 1 [file 44318_2024_101_MOESM2_ESM.zip › Figure 1/1I/western MT-ATP6.tif]

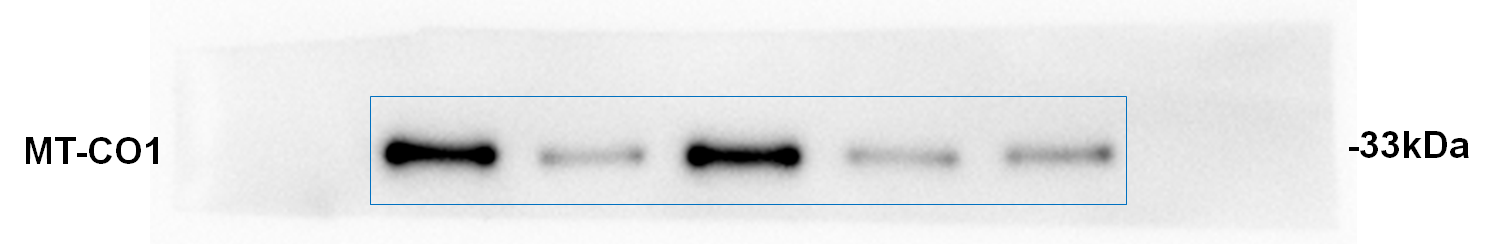

Supplement: Supplementary file 2 — Source data Fig. 1 [file 44318_2024_101_MOESM2_ESM.zip › Figure 1/1I/western MT-CO1.tif]

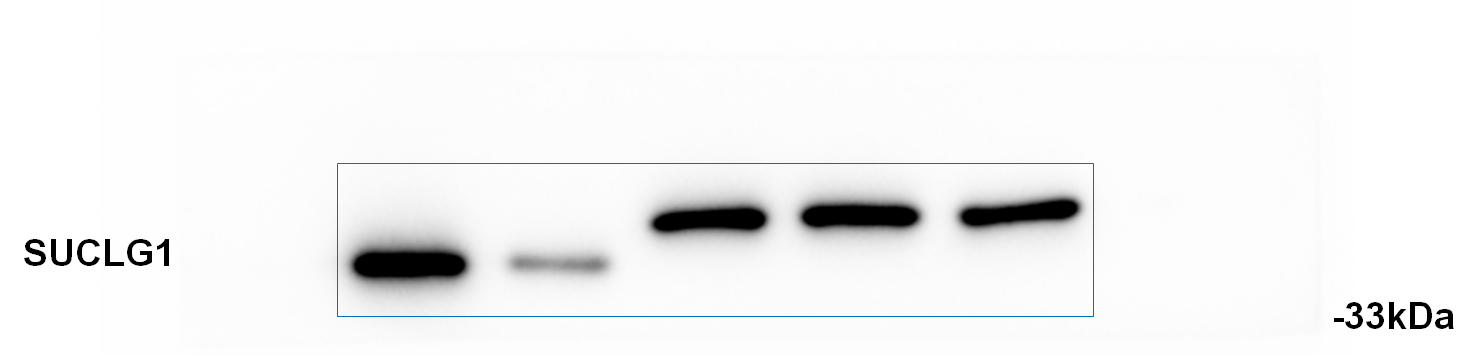

Supplement: Supplementary file 2 — Source data Fig. 1 [file 44318_2024_101_MOESM2_ESM.zip › Figure 1/1I/western SUCLG1.tif]

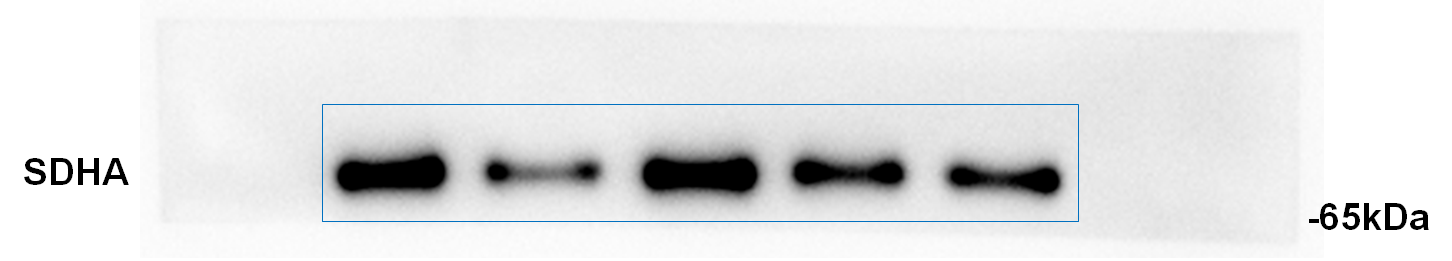

Supplement: Supplementary file 2 — Source data Fig. 1 [file 44318_2024_101_MOESM2_ESM.zip › Figure 1/1I/western SDHA.tif]

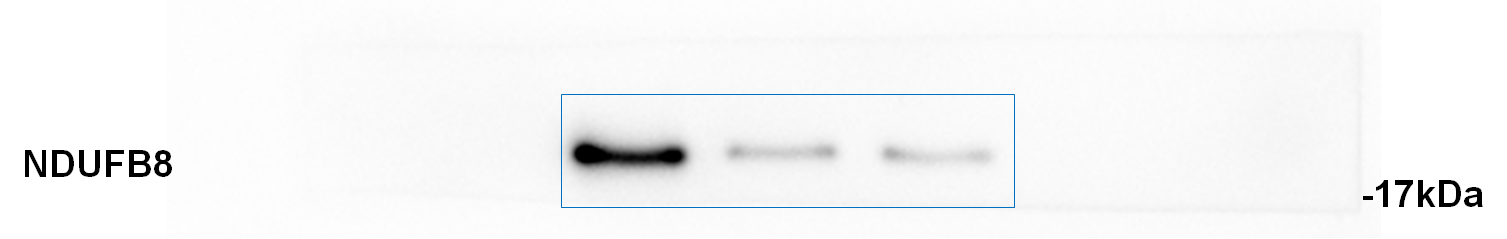

Supplement: Supplementary file 2 — Source data Fig. 1 [file 44318_2024_101_MOESM2_ESM.zip › Figure 1/1E/western MV411 NDUFB8.tif]

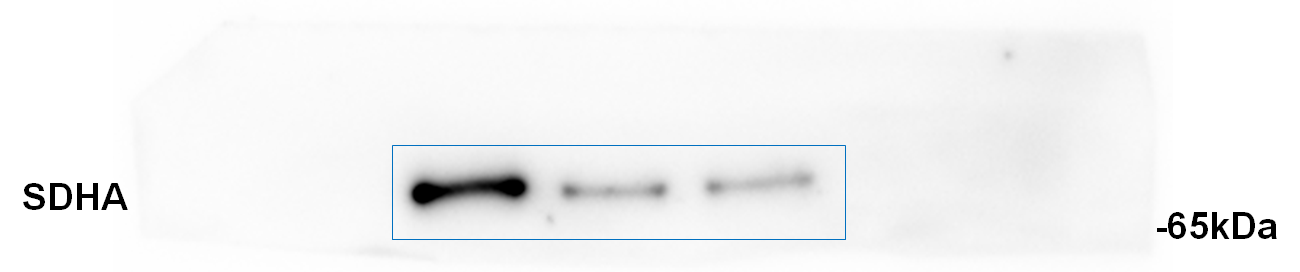

Supplement: Supplementary file 2 — Source data Fig. 1 [file 44318_2024_101_MOESM2_ESM.zip › Figure 1/1E/western HL60 SDHA.tif]

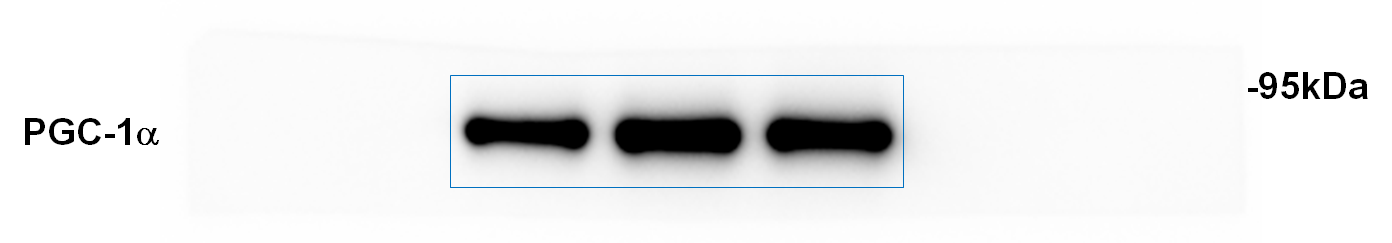

Supplement: Supplementary file 2 — Source data Fig. 1 [file 44318_2024_101_MOESM2_ESM.zip › Figure 1/1E/western MV411 PGC1a.tif]

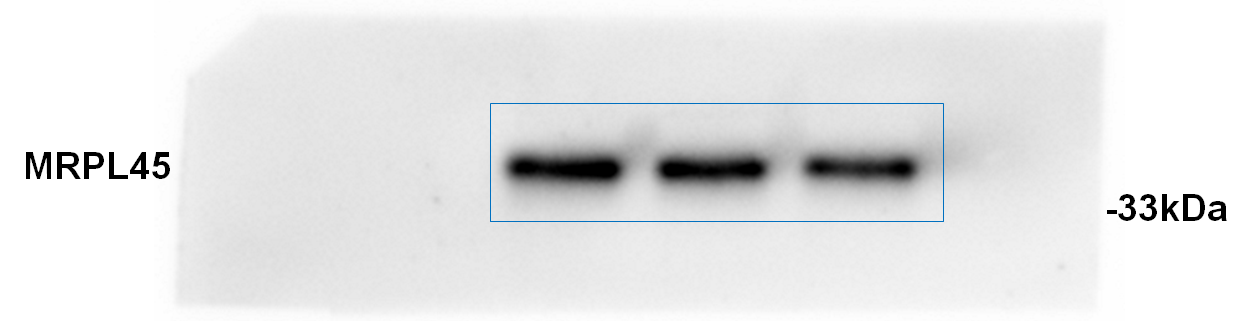

Supplement: Supplementary file 2 — Source data Fig. 1 [file 44318_2024_101_MOESM2_ESM.zip › Figure 1/1E/western MV411 MRPL45.tif]

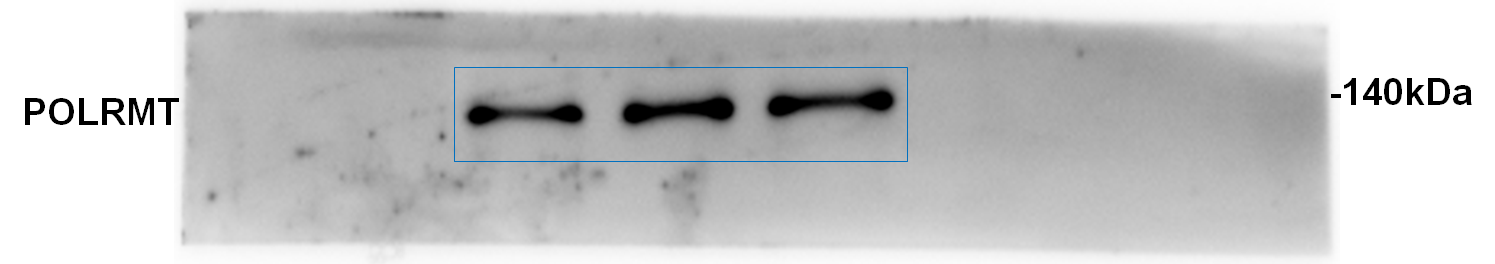

Supplement: Supplementary file 2 — Source data Fig. 1 [file 44318_2024_101_MOESM2_ESM.zip › Figure 1/1E/western HL60 POLRMT.tif]

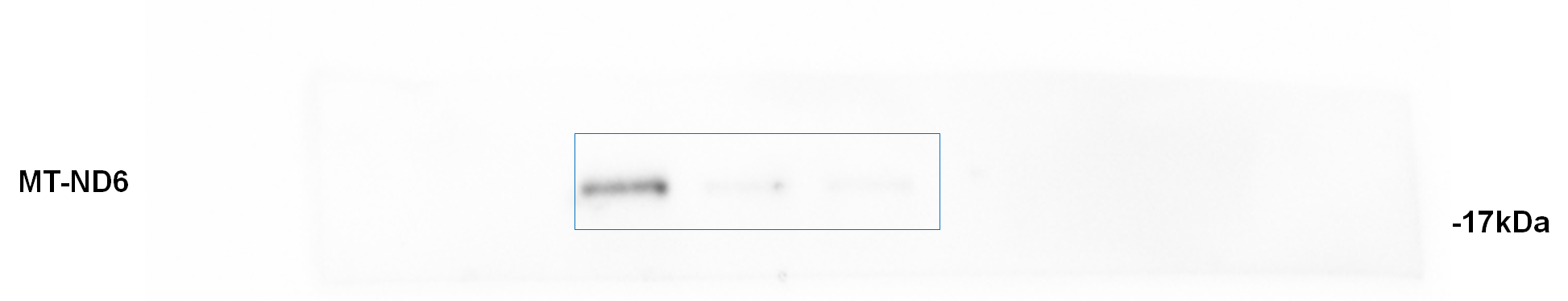

Supplement: Supplementary file 2 — Source data Fig. 1 [file 44318_2024_101_MOESM2_ESM.zip › Figure 1/1E/western HL60 MT-ND6.tif]

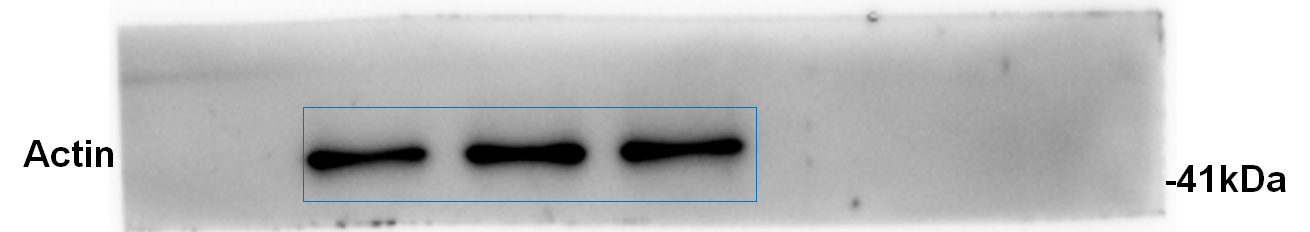

Supplement: Supplementary file 2 — Source data Fig. 1 [file 44318_2024_101_MOESM2_ESM.zip › Figure 1/1E/western HL60 Actin.tif]

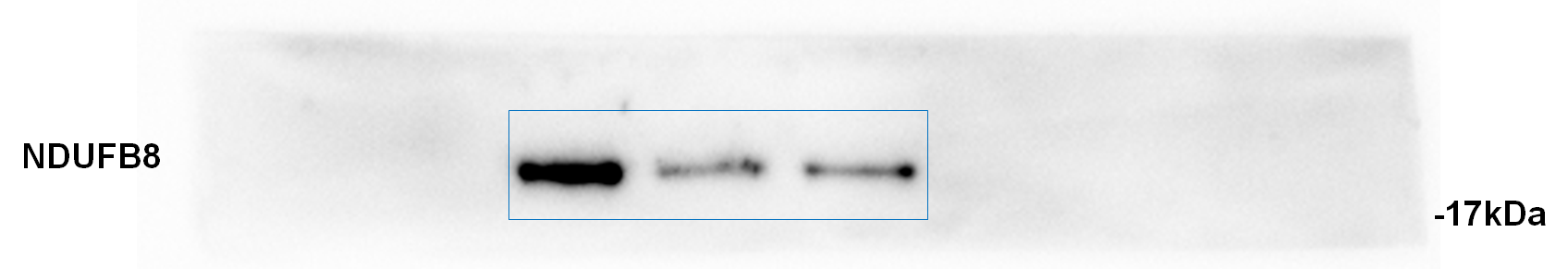

Supplement: Supplementary file 2 — Source data Fig. 1 [file 44318_2024_101_MOESM2_ESM.zip › Figure 1/1E/western HL60 NDUFB8.tif]

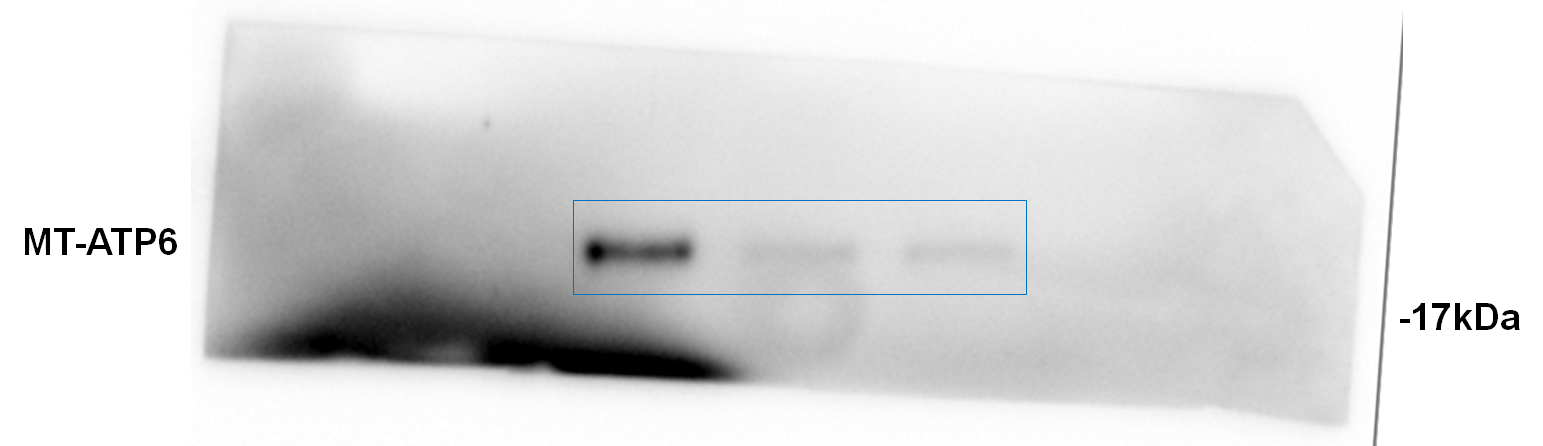

Supplement: Supplementary file 2 — Source data Fig. 1 [file 44318_2024_101_MOESM2_ESM.zip › Figure 1/1E/western HL60 MT-ATP6.tif]

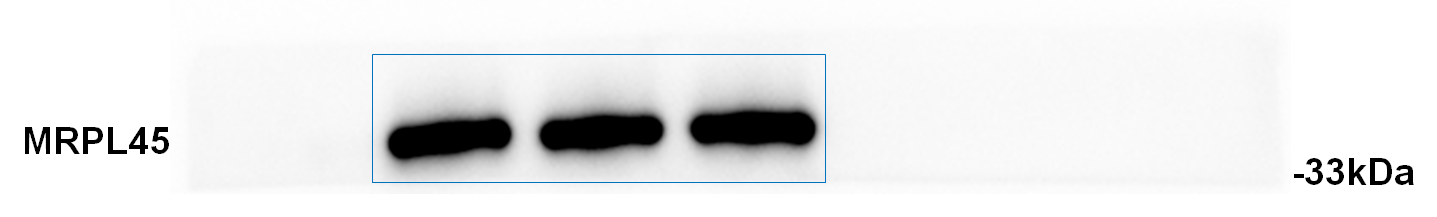

Supplement: Supplementary file 2 — Source data Fig. 1 [file 44318_2024_101_MOESM2_ESM.zip › Figure 1/1E/western HL60 MRPL45.tif]

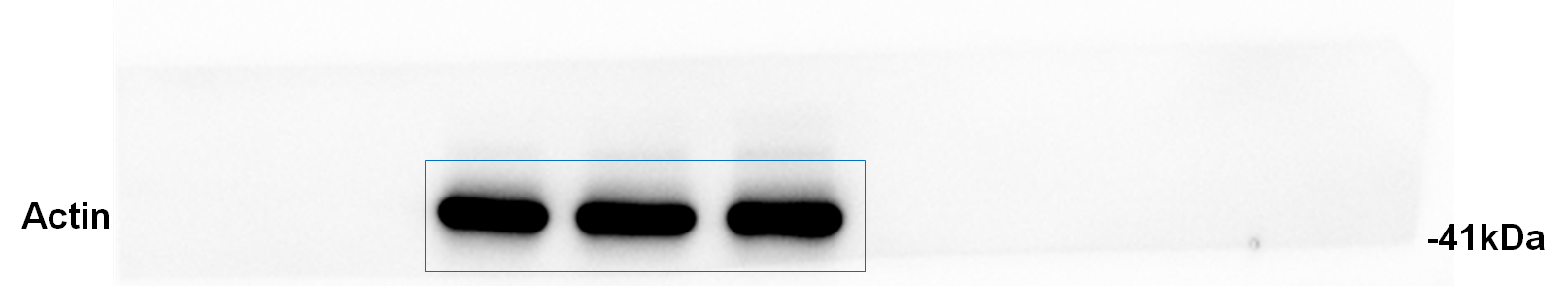

Supplement: Supplementary file 2 — Source data Fig. 1 [file 44318_2024_101_MOESM2_ESM.zip › Figure 1/1E/western MV411 Actin.tif]

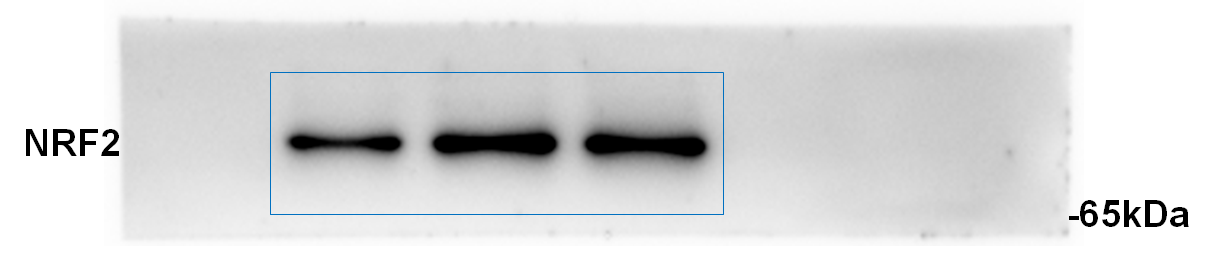

Supplement: Supplementary file 2 — Source data Fig. 1 [file 44318_2024_101_MOESM2_ESM.zip › Figure 1/1E/western MV411 NRF2.tif]

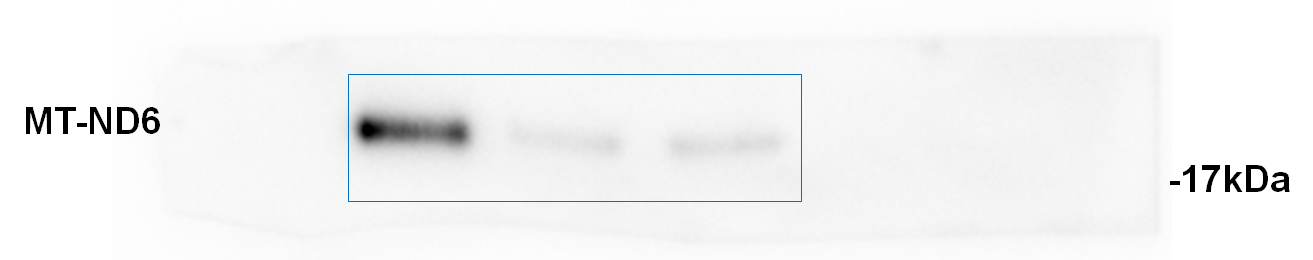

Supplement: Supplementary file 2 — Source data Fig. 1 [file 44318_2024_101_MOESM2_ESM.zip › Figure 1/1E/western MV411 MT-ND6.tif]

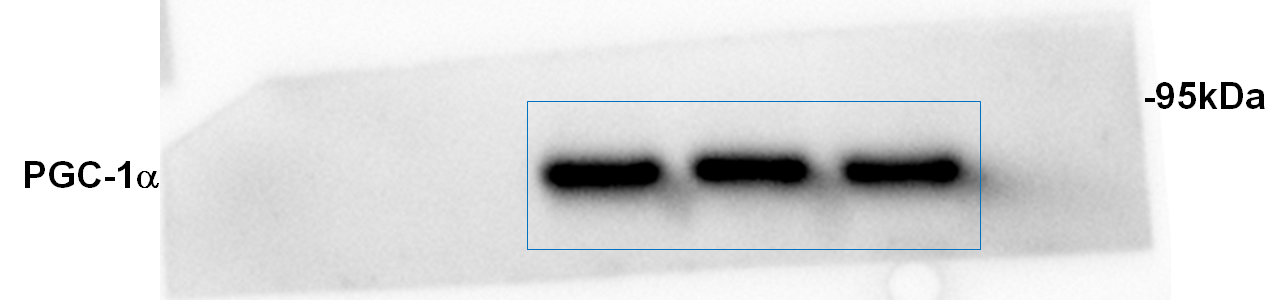

Supplement: Supplementary file 2 — Source data Fig. 1 [file 44318_2024_101_MOESM2_ESM.zip › Figure 1/1E/western HL60 PGC1a.tif]

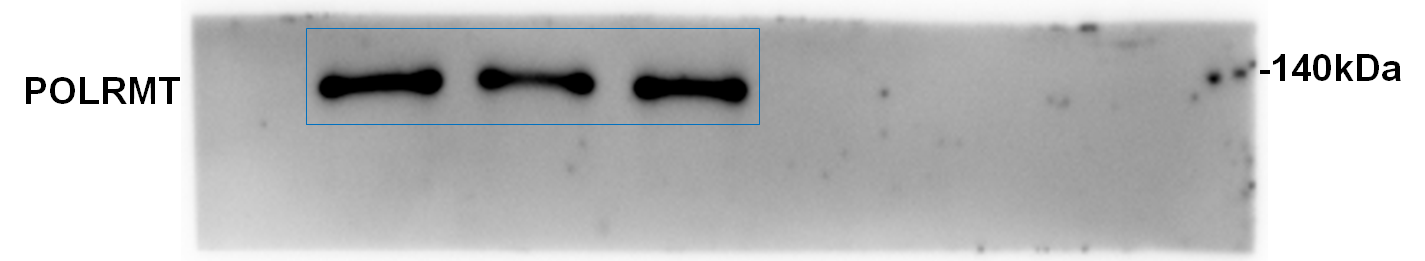

Supplement: Supplementary file 2 — Source data Fig. 1 [file 44318_2024_101_MOESM2_ESM.zip › Figure 1/1E/western MV411 POLRMT.tif]

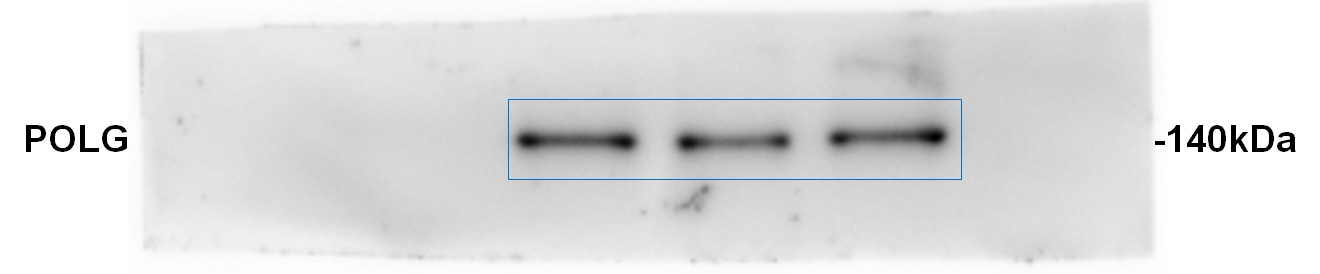

Supplement: Supplementary file 2 — Source data Fig. 1 [file 44318_2024_101_MOESM2_ESM.zip › Figure 1/1E/western MV411 POLG.tif]

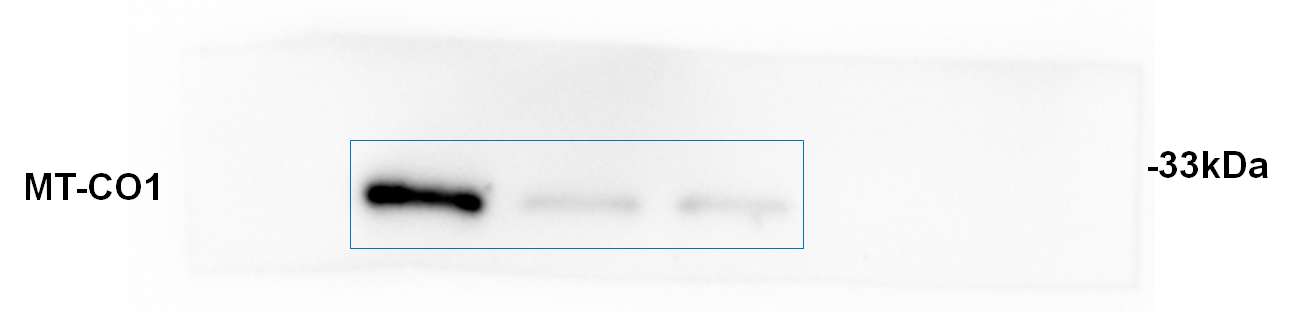

Supplement: Supplementary file 2 — Source data Fig. 1 [file 44318_2024_101_MOESM2_ESM.zip › Figure 1/1E/western MV411 MT-CO1.tif]

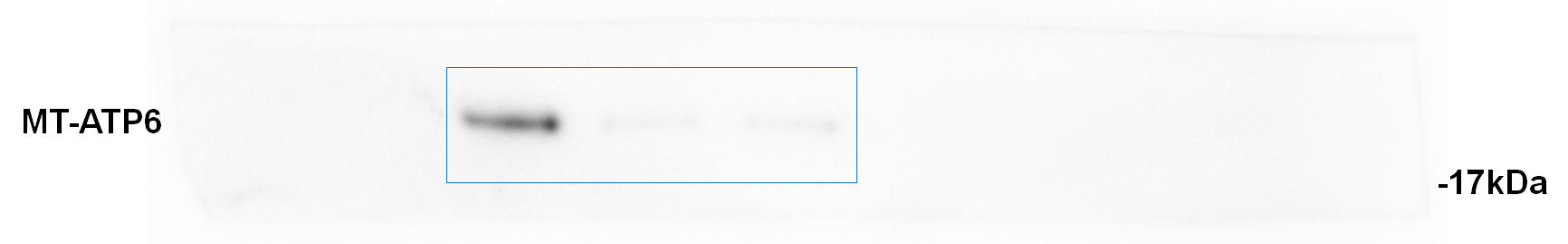

Supplement: Supplementary file 2 — Source data Fig. 1 [file 44318_2024_101_MOESM2_ESM.zip › Figure 1/1E/western MV411 MT-ATP6.tif]

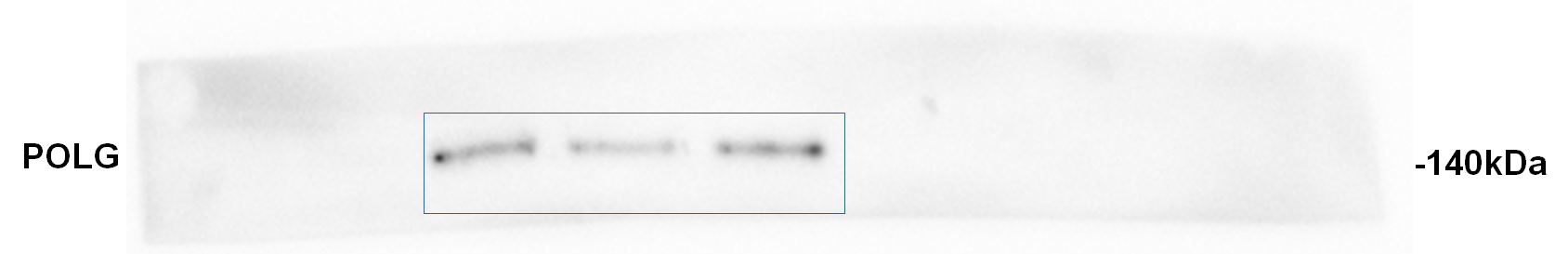

Supplement: Supplementary file 2 — Source data Fig. 1 [file 44318_2024_101_MOESM2_ESM.zip › Figure 1/1E/western HL60 POLG.tif]

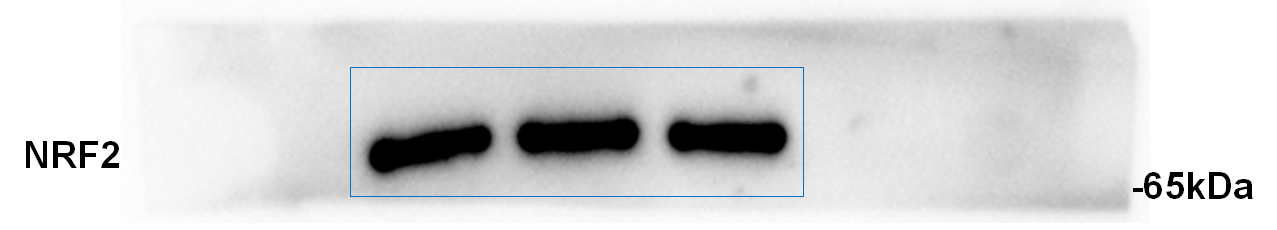

Supplement: Supplementary file 2 — Source data Fig. 1 [file 44318_2024_101_MOESM2_ESM.zip › Figure 1/1E/western HL60 NRF2.tif]

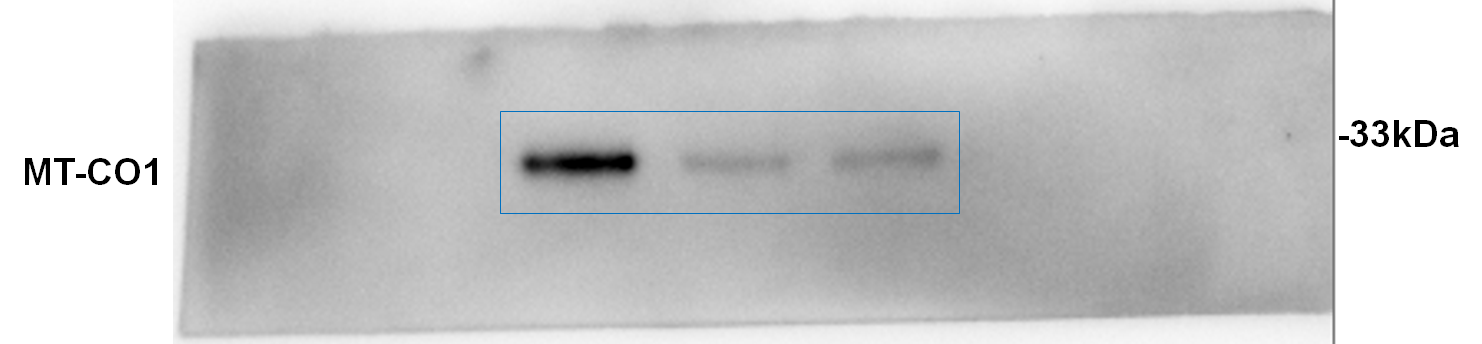

Supplement: Supplementary file 2 — Source data Fig. 1 [file 44318_2024_101_MOESM2_ESM.zip › Figure 1/1E/western HL60 MT-CO1.tif]

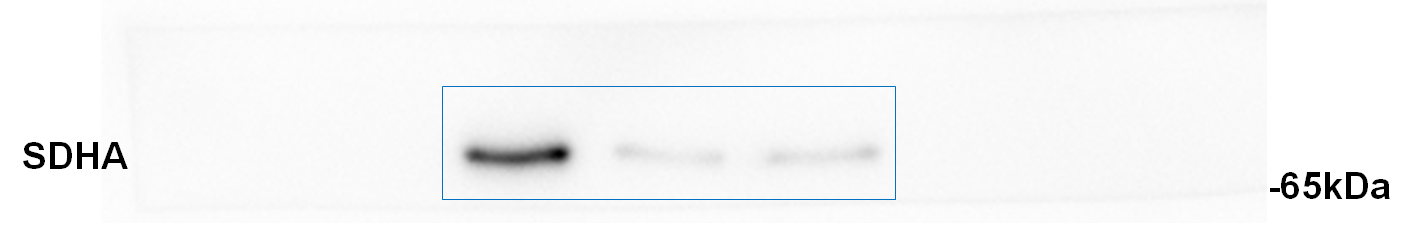

Supplement: Supplementary file 2 — Source data Fig. 1 [file 44318_2024_101_MOESM2_ESM.zip › Figure 1/1E/western MV411 SDHA.tif]

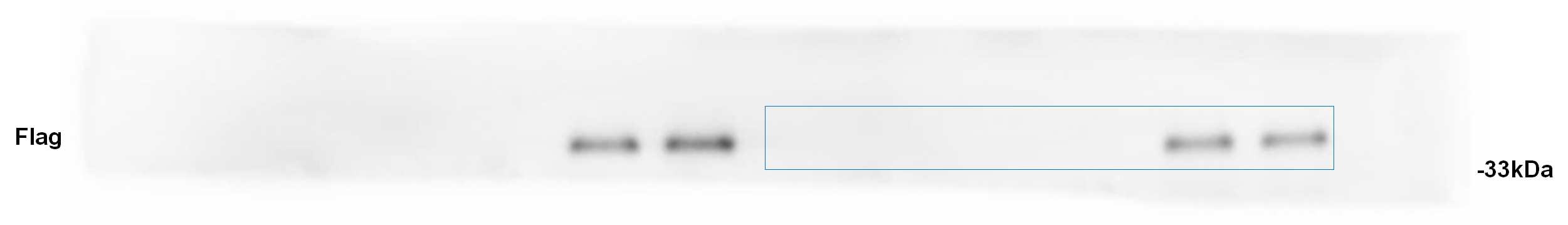

Supplement: Supplementary file 3 — Source data Fig. 2 [file 44318_2024_101_MOESM3_ESM.zip › Figure 2/2N/western Flag.tif]

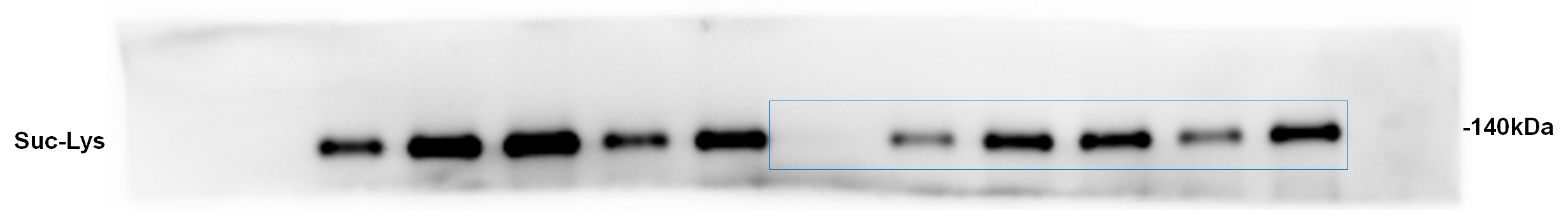

Supplement: Supplementary file 3 — Source data Fig. 2 [file 44318_2024_101_MOESM3_ESM.zip › Figure 2/2N/western suc-Lys.tif]

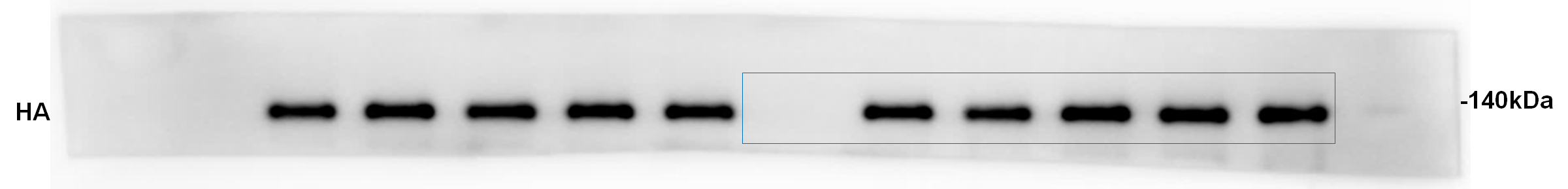

Supplement: Supplementary file 3 — Source data Fig. 2 [file 44318_2024_101_MOESM3_ESM.zip › Figure 2/2N/western HA.tif]

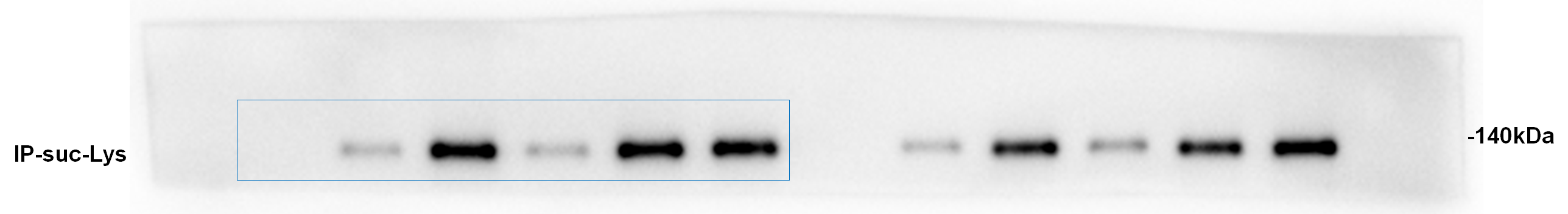

Supplement: Supplementary file 3 — Source data Fig. 2 [file 44318_2024_101_MOESM3_ESM.zip › Figure 2/2O/western IP-suc-Lys.tif]

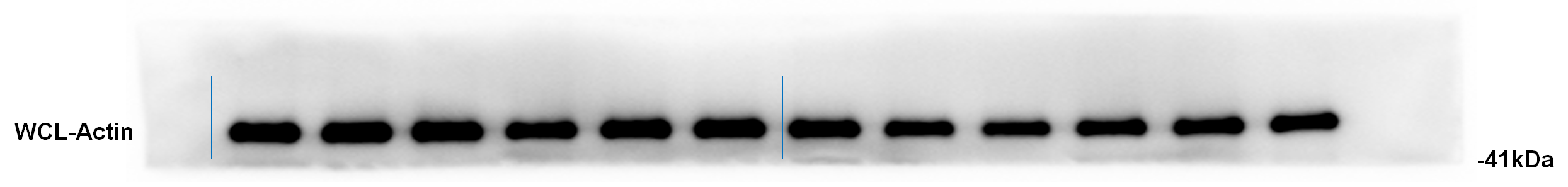

Supplement: Supplementary file 3 — Source data Fig. 2 [file 44318_2024_101_MOESM3_ESM.zip › Figure 2/2O/western WCL-Actin.tif]

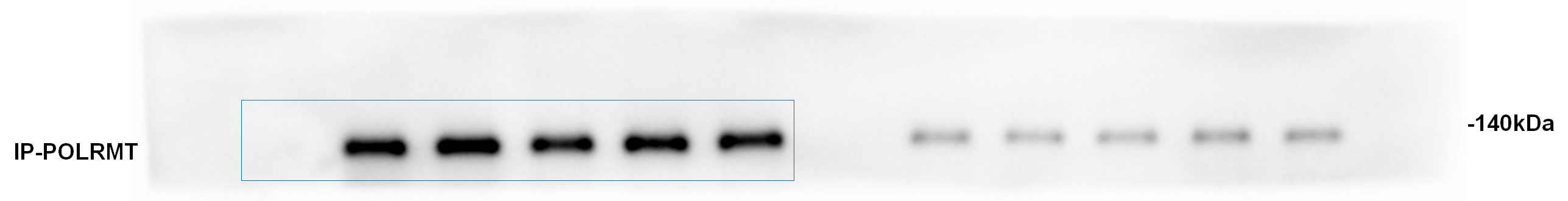

Supplement: Supplementary file 3 — Source data Fig. 2 [file 44318_2024_101_MOESM3_ESM.zip › Figure 2/2O/western IP-POLRMT.tif]

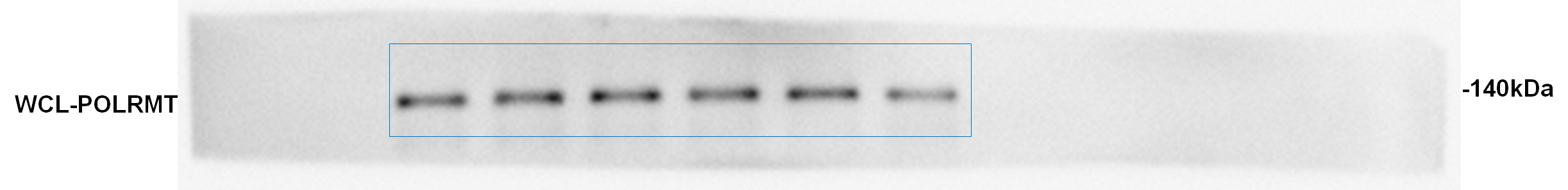

Supplement: Supplementary file 3 — Source data Fig. 2 [file 44318_2024_101_MOESM3_ESM.zip › Figure 2/2O/western WCL-POLRMT.tif]

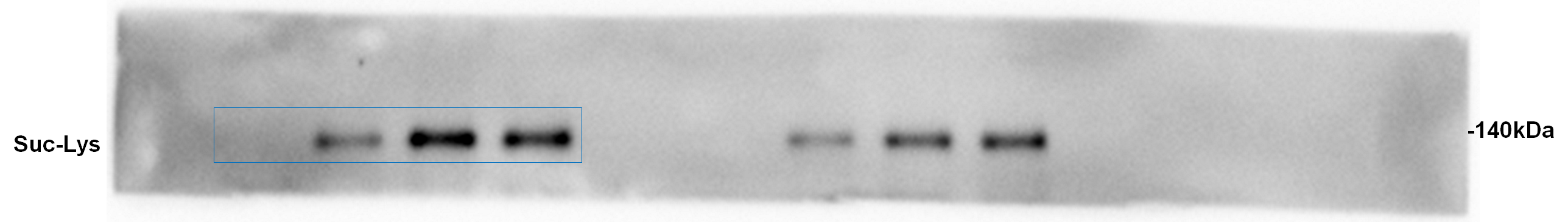

Supplement: Supplementary file 3 — Source data Fig. 2 [file 44318_2024_101_MOESM3_ESM.zip › Figure 2/2M/western suc-Lys.tif]

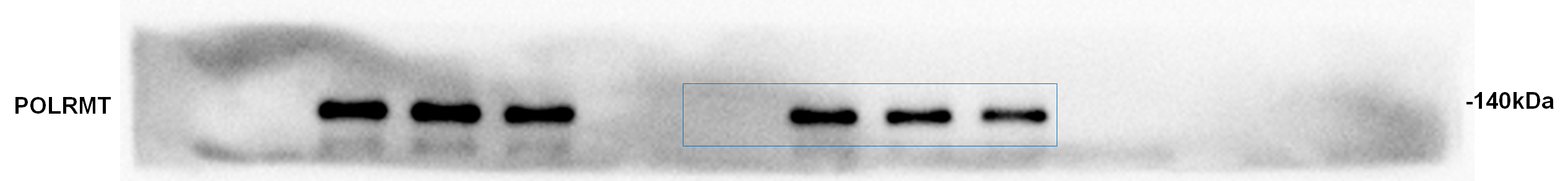

Supplement: Supplementary file 3 — Source data Fig. 2 [file 44318_2024_101_MOESM3_ESM.zip › Figure 2/2M/western POLRMT.tif]

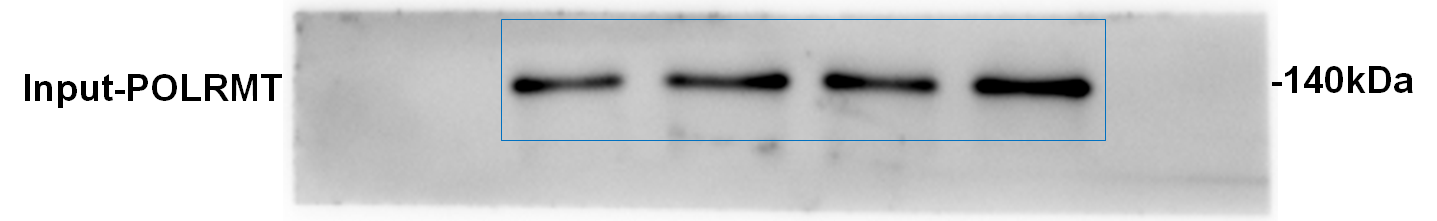

Supplement: Supplementary file 3 — Source data Fig. 2 [file 44318_2024_101_MOESM3_ESM.zip › Figure 2/2K/western Input POLRMT.tif]

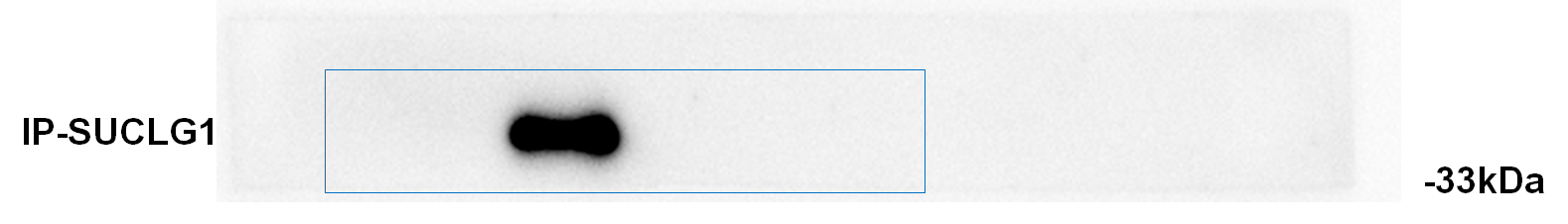

Supplement: Supplementary file 3 — Source data Fig. 2 [file 44318_2024_101_MOESM3_ESM.zip › Figure 2/2K/western IP-SUCLG1.tif]

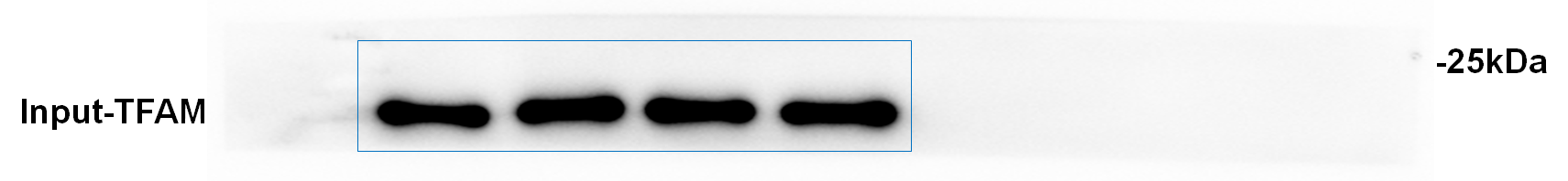

Supplement: Supplementary file 3 — Source data Fig. 2 [file 44318_2024_101_MOESM3_ESM.zip › Figure 2/2K/western Input TFAM.tif]

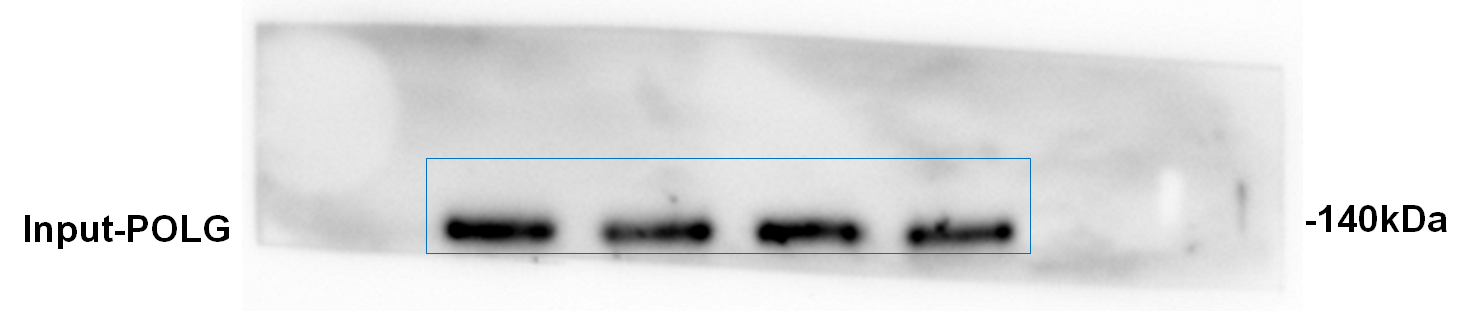

Supplement: Supplementary file 3 — Source data Fig. 2 [file 44318_2024_101_MOESM3_ESM.zip › Figure 2/2K/western Input POLG.tif]

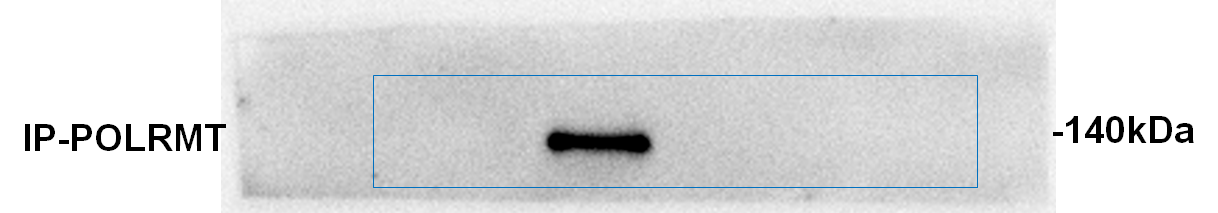

Supplement: Supplementary file 3 — Source data Fig. 2 [file 44318_2024_101_MOESM3_ESM.zip › Figure 2/2K/western IP-POLRMT.tif]

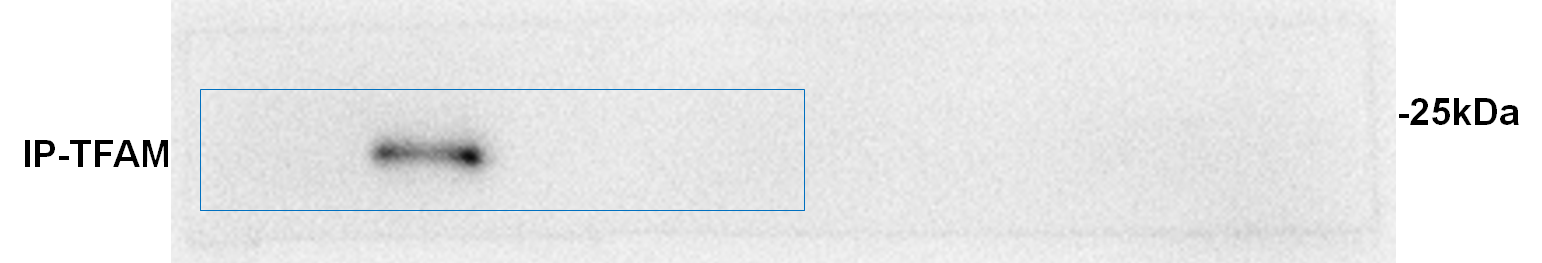

Supplement: Supplementary file 3 — Source data Fig. 2 [file 44318_2024_101_MOESM3_ESM.zip › Figure 2/2K/western IP-TFAM.tif]

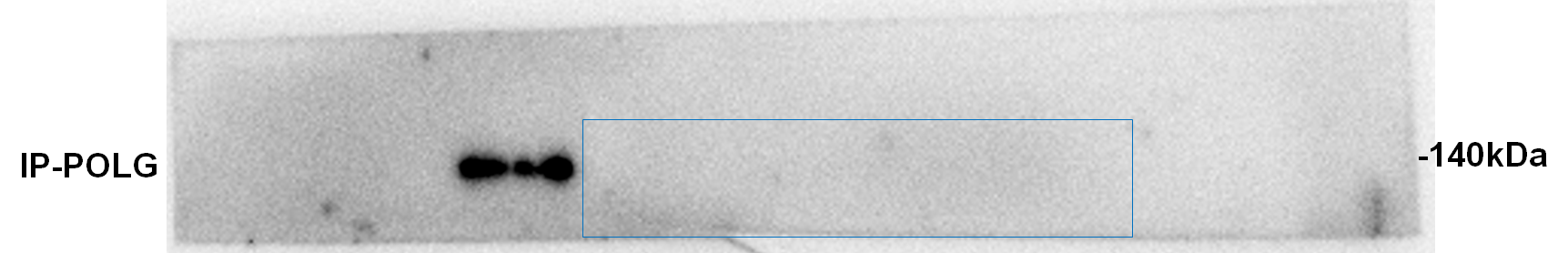

Supplement: Supplementary file 3 — Source data Fig. 2 [file 44318_2024_101_MOESM3_ESM.zip › Figure 2/2K/western IP-POLG.tif]

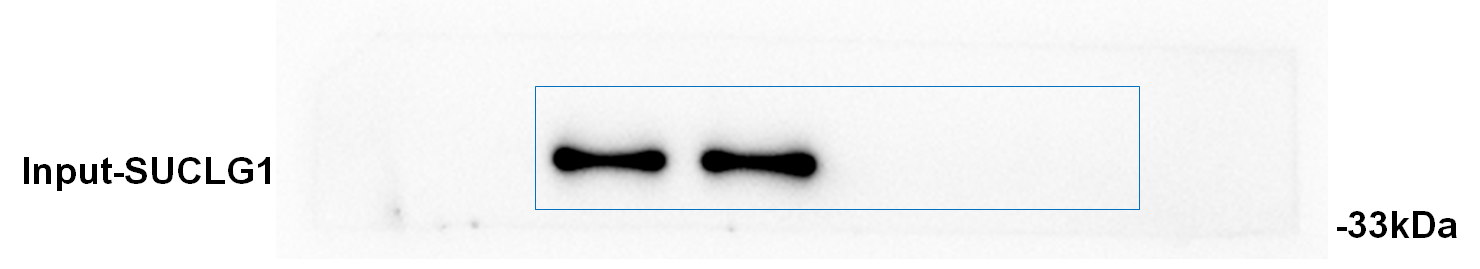

Supplement: Supplementary file 3 — Source data Fig. 2 [file 44318_2024_101_MOESM3_ESM.zip › Figure 2/2K/western Input SUCLG1.tif]

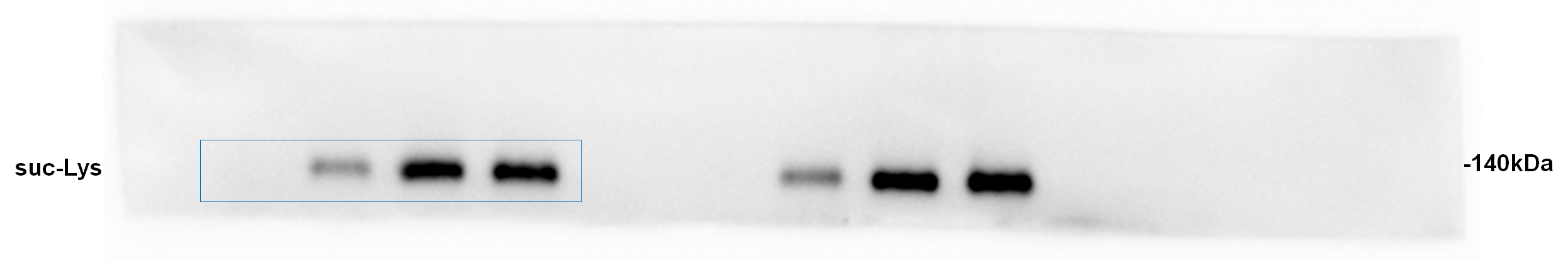

Supplement: Supplementary file 3 — Source data Fig. 2 [file 44318_2024_101_MOESM3_ESM.zip › Figure 2/2L/western suc-Lys.tif]

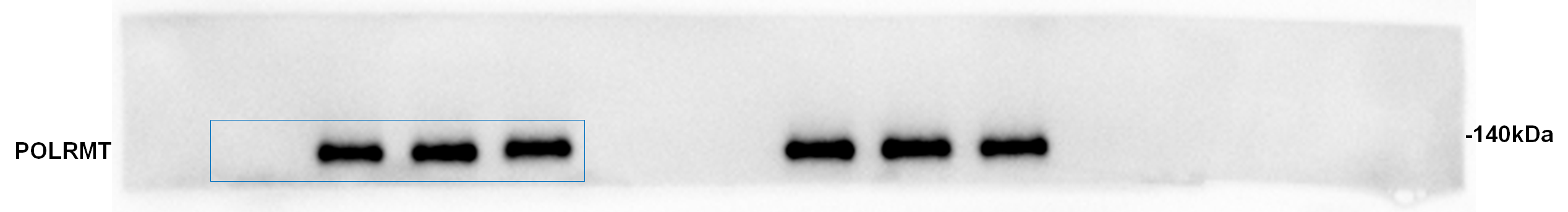

Supplement: Supplementary file 3 — Source data Fig. 2 [file 44318_2024_101_MOESM3_ESM.zip › Figure 2/2L/western POLRMT.tif]

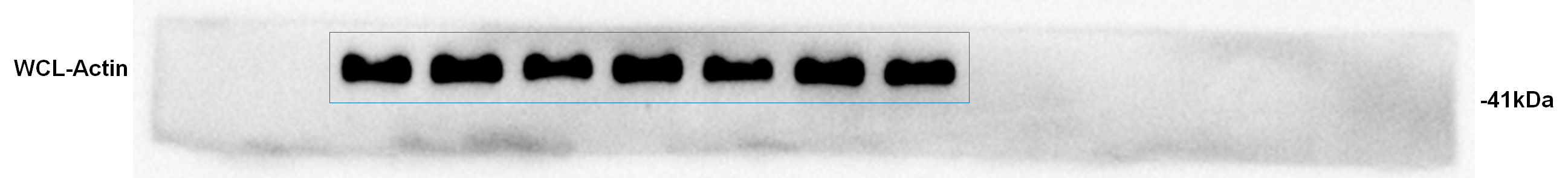

Supplement: Supplementary file 4 — Source data Fig. 3 [file 44318_2024_101_MOESM4_ESM.zip › Figure 3/3B/western WCL-Actin.tif]

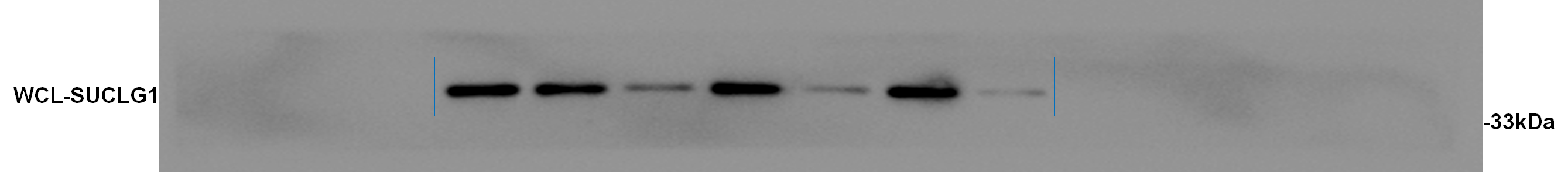

Supplement: Supplementary file 4 — Source data Fig. 3 [file 44318_2024_101_MOESM4_ESM.zip › Figure 3/3B/western WCL-SUCLG1.tif]

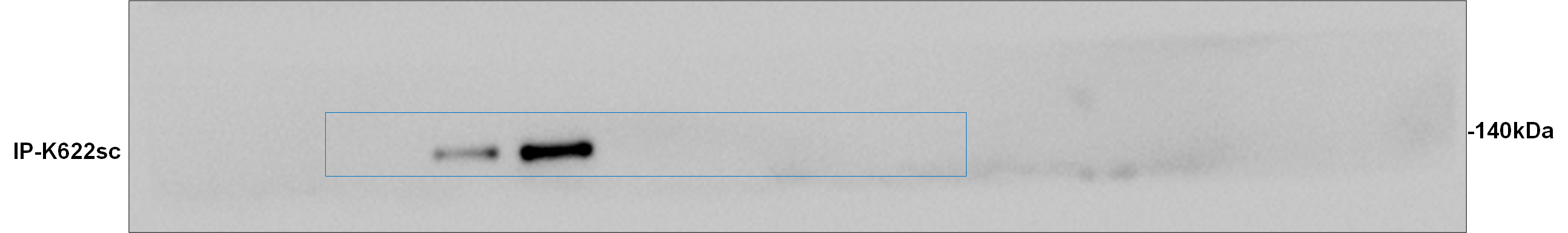

Supplement: Supplementary file 4 — Source data Fig. 3 [file 44318_2024_101_MOESM4_ESM.zip › Figure 3/3B/western IP-K622sc.tif]

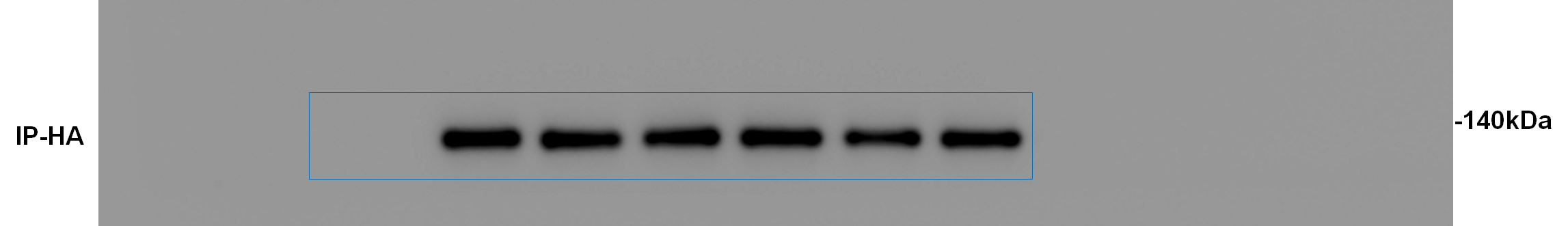

Supplement: Supplementary file 4 — Source data Fig. 3 [file 44318_2024_101_MOESM4_ESM.zip › Figure 3/3B/western IP-HA.tif]

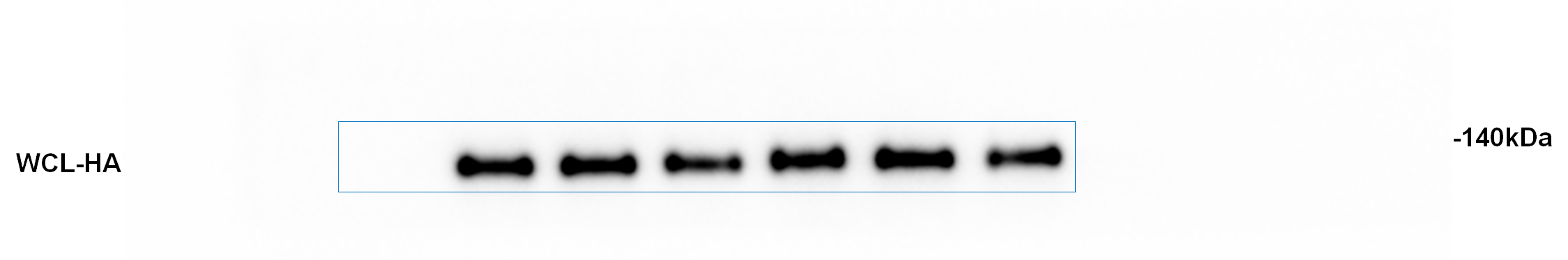

Supplement: Supplementary file 4 — Source data Fig. 3 [file 44318_2024_101_MOESM4_ESM.zip › Figure 3/3B/western WCL-HA.tif]

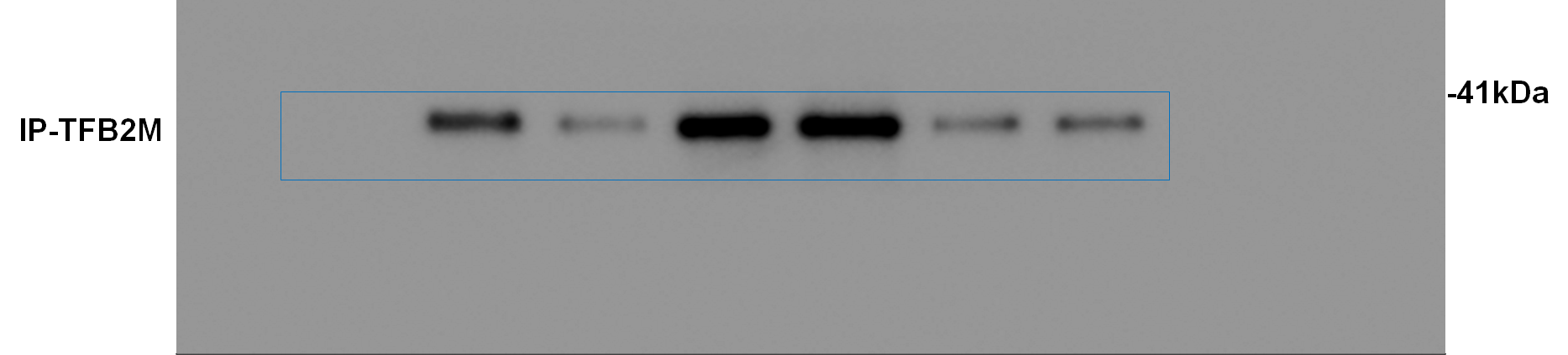

Supplement: Supplementary file 4 — Source data Fig. 3 [file 44318_2024_101_MOESM4_ESM.zip › Figure 3/3B/western IP-TFB2M.tif]

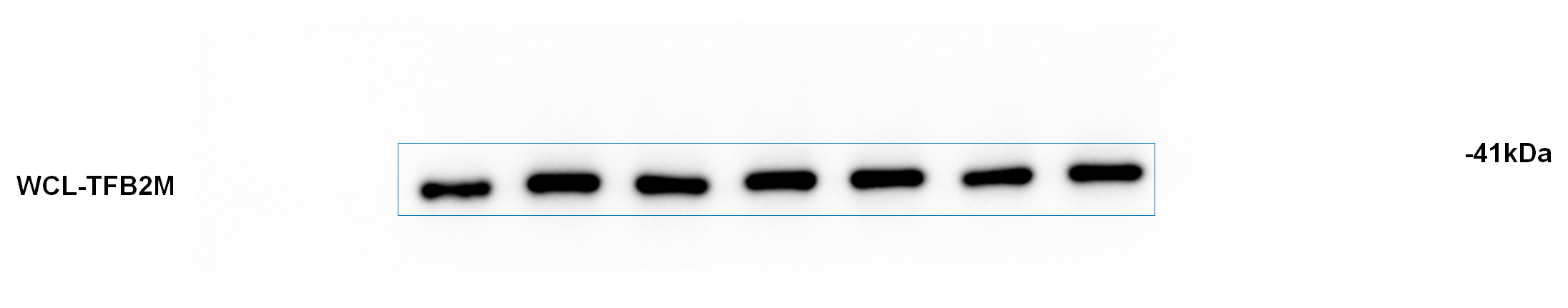

Supplement: Supplementary file 4 — Source data Fig. 3 [file 44318_2024_101_MOESM4_ESM.zip › Figure 3/3B/western WCL-TFB2M.tif]

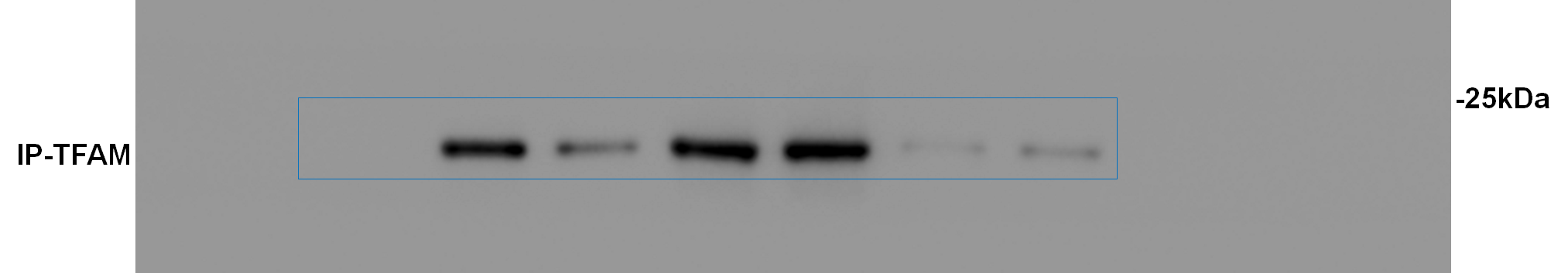

Supplement: Supplementary file 4 — Source data Fig. 3 [file 44318_2024_101_MOESM4_ESM.zip › Figure 3/3B/western IP-TFAM.tif]

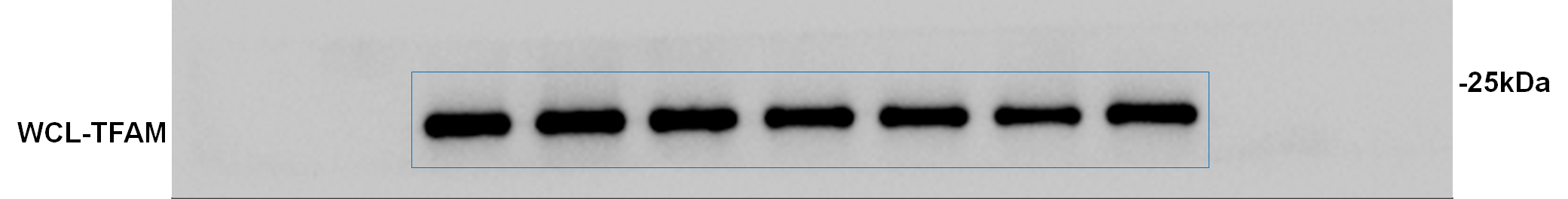

Supplement: Supplementary file 4 — Source data Fig. 3 [file 44318_2024_101_MOESM4_ESM.zip › Figure 3/3B/western WCL-TFAM.tif]

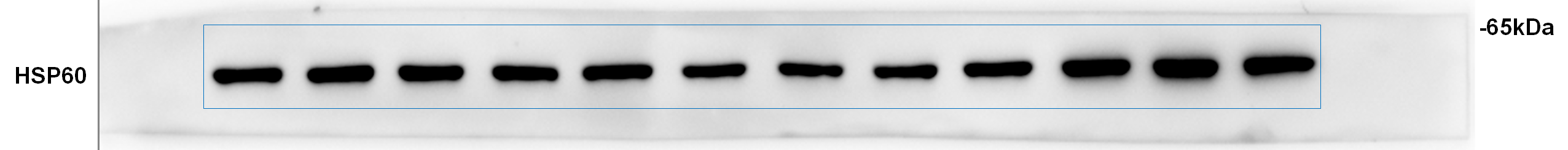

Supplement: Supplementary file 4 — Source data Fig. 3 [file 44318_2024_101_MOESM4_ESM.zip › Figure 3/3D/western HSP60.tif]

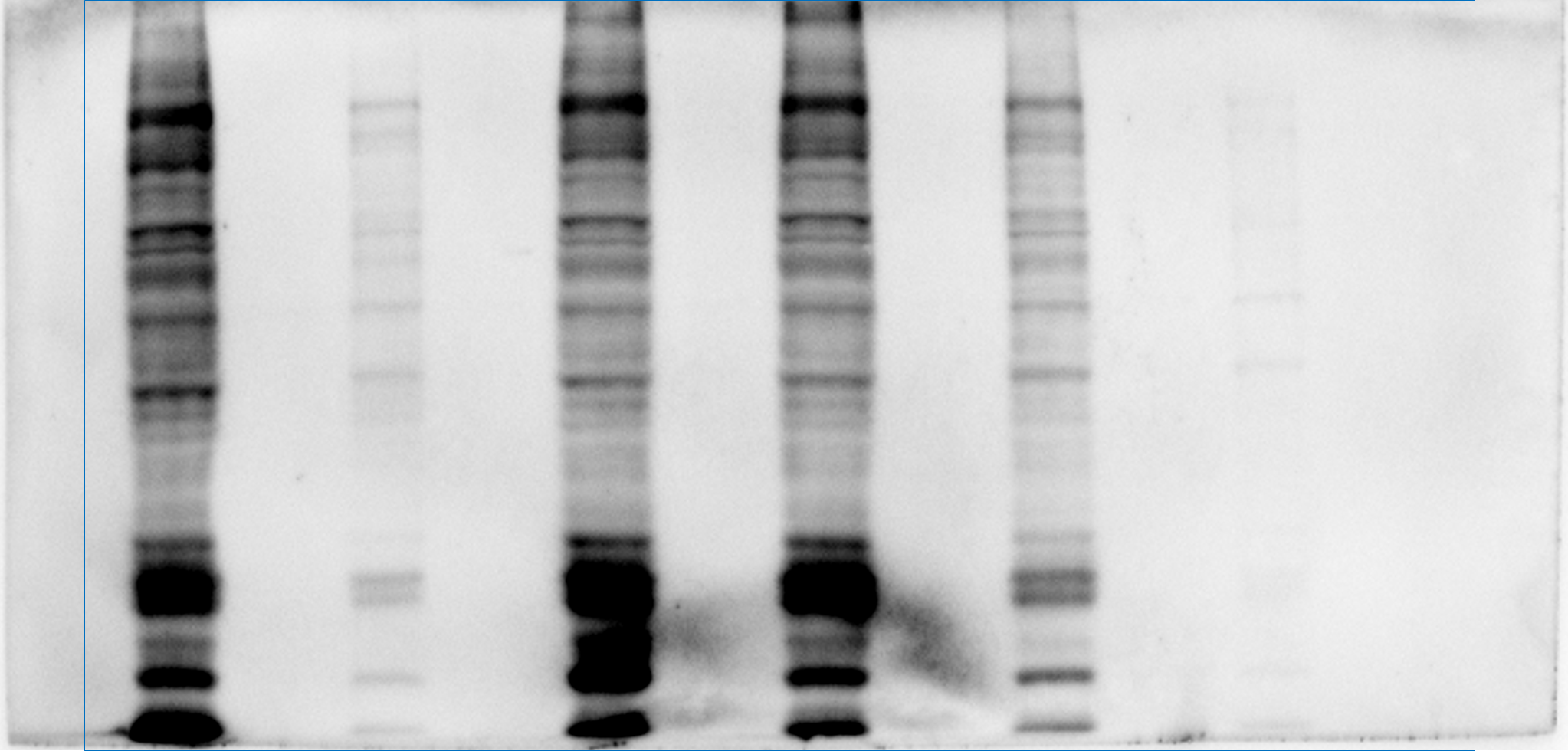

Supplement: Supplementary file 4 — Source data Fig. 3 [file 44318_2024_101_MOESM4_ESM.zip › Figure 3/3D/UTP labeling.tif]

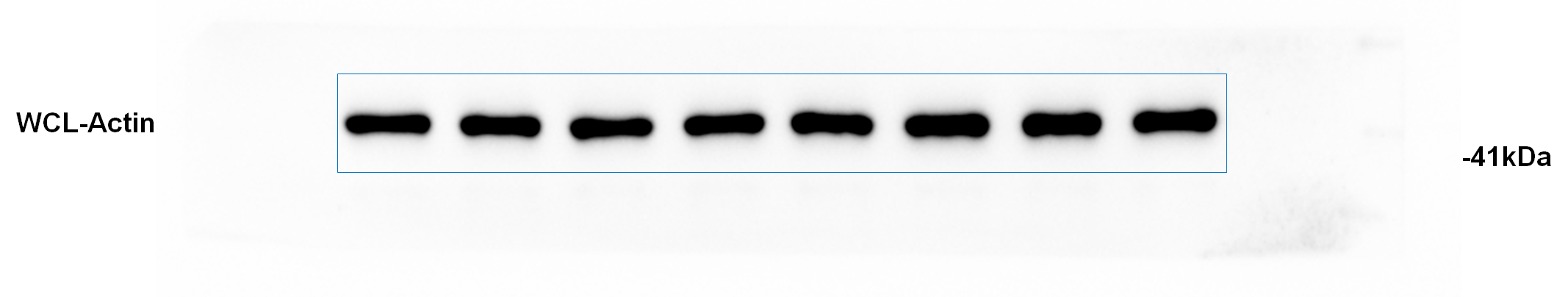

Supplement: Supplementary file 4 — Source data Fig. 3 [file 44318_2024_101_MOESM4_ESM.zip › Figure 3/3O/western WCL-Actin.tif]

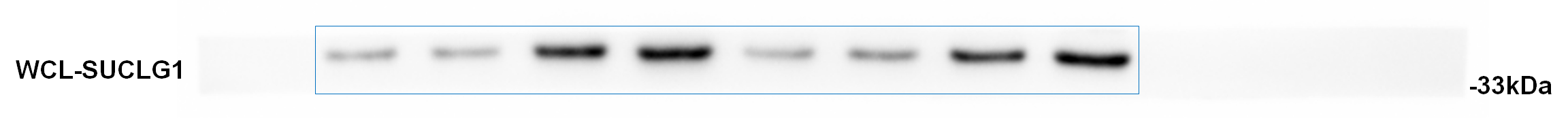

Supplement: Supplementary file 4 — Source data Fig. 3 [file 44318_2024_101_MOESM4_ESM.zip › Figure 3/3O/western WCL-SUCLG1.tif]

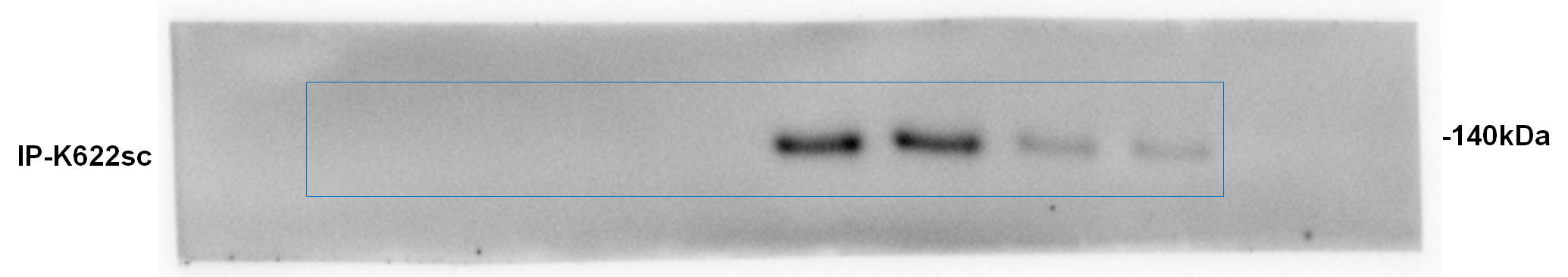

Supplement: Supplementary file 4 — Source data Fig. 3 [file 44318_2024_101_MOESM4_ESM.zip › Figure 3/3O/western IP-K622sc.tif]

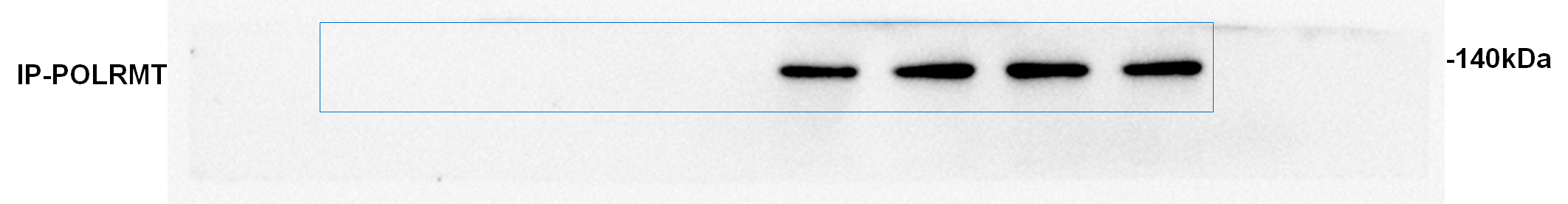

Supplement: Supplementary file 4 — Source data Fig. 3 [file 44318_2024_101_MOESM4_ESM.zip › Figure 3/3O/western IP-POLRMT.tif]

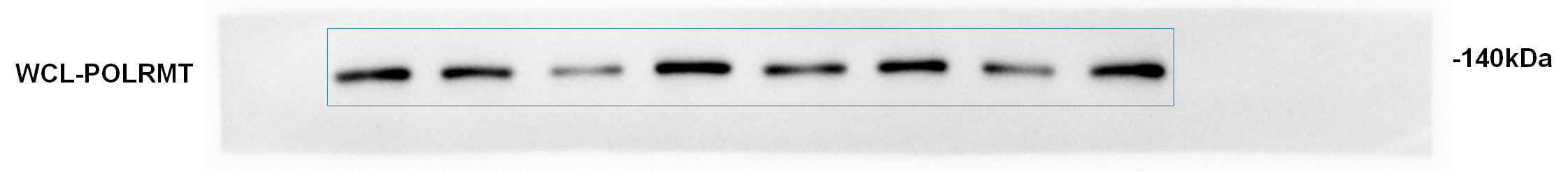

Supplement: Supplementary file 4 — Source data Fig. 3 [file 44318_2024_101_MOESM4_ESM.zip › Figure 3/3O/western WCL-POLRMT.tif]

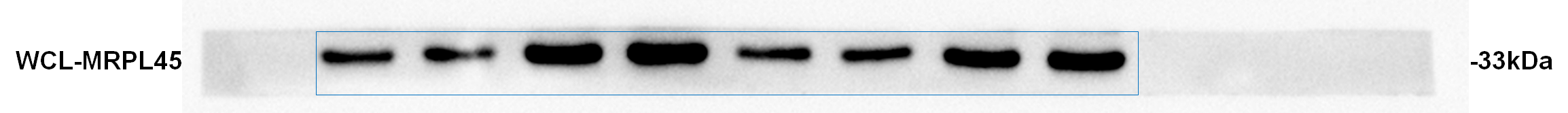

Supplement: Supplementary file 4 — Source data Fig. 3 [file 44318_2024_101_MOESM4_ESM.zip › Figure 3/3O/western WCL-MRPL45.tif]

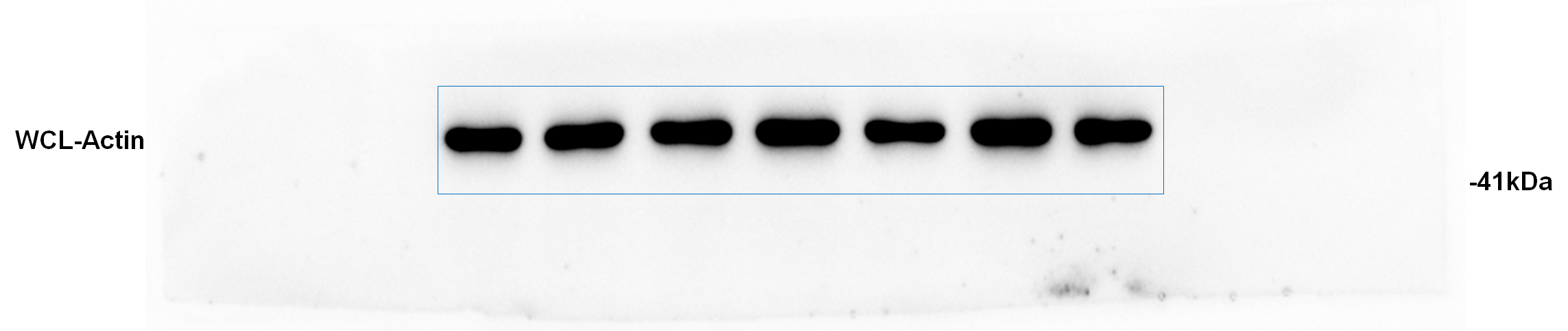

Supplement: Supplementary file 4 — Source data Fig. 3 [file 44318_2024_101_MOESM4_ESM.zip › Figure 3/3I/western WCL-Actin.tif]

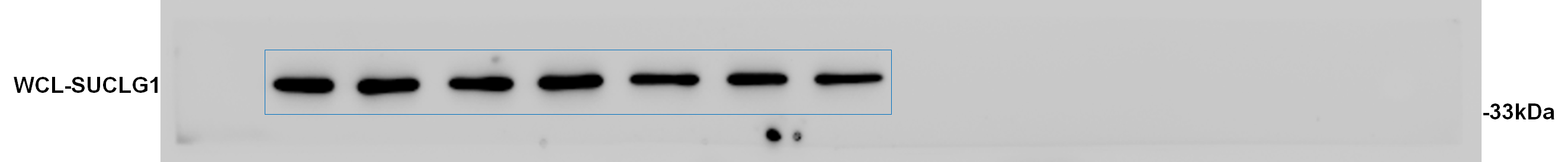

Supplement: Supplementary file 4 — Source data Fig. 3 [file 44318_2024_101_MOESM4_ESM.zip › Figure 3/3I/western WCL-SUCLG1.tif]

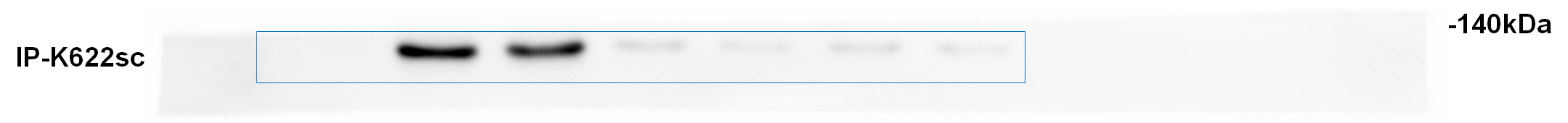

Supplement: Supplementary file 4 — Source data Fig. 3 [file 44318_2024_101_MOESM4_ESM.zip › Figure 3/3I/western IP-K622sc.tif]

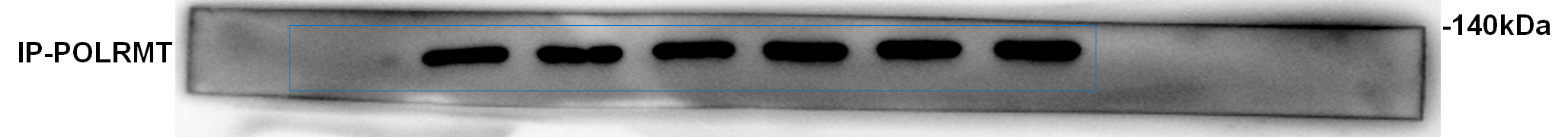

Supplement: Supplementary file 4 — Source data Fig. 3 [file 44318_2024_101_MOESM4_ESM.zip › Figure 3/3I/western IP-POLRMT.tif]

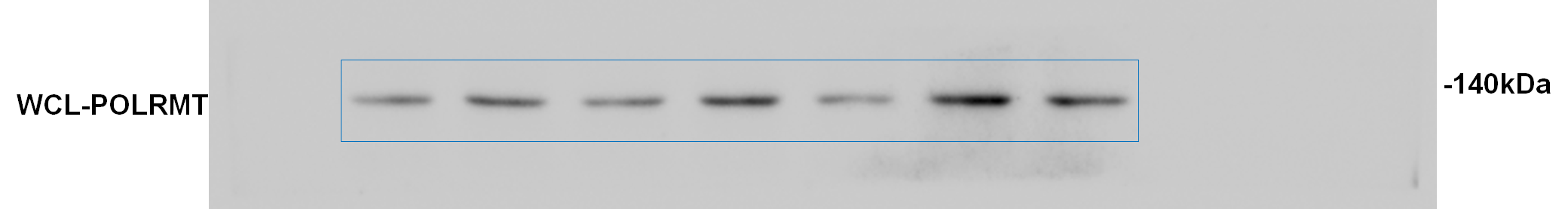

Supplement: Supplementary file 4 — Source data Fig. 3 [file 44318_2024_101_MOESM4_ESM.zip › Figure 3/3I/western WCL-POLRMT.tif]

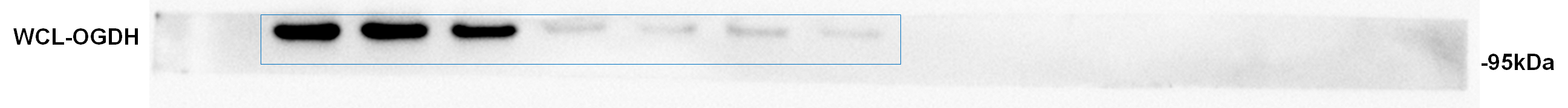

Supplement: Supplementary file 4 — Source data Fig. 3 [file 44318_2024_101_MOESM4_ESM.zip › Figure 3/3I/western WCL-OGDH.tif]

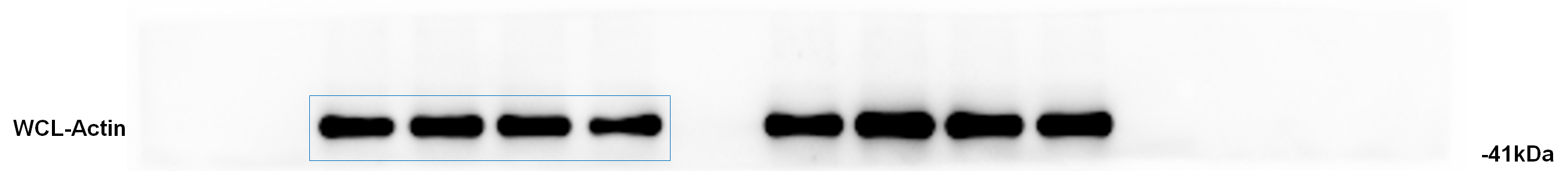

Supplement: Supplementary file 5 — Source data Fig. 4 [file 44318_2024_101_MOESM5_ESM.zip › Figure 4/4L/western WCL-Actin.tif]

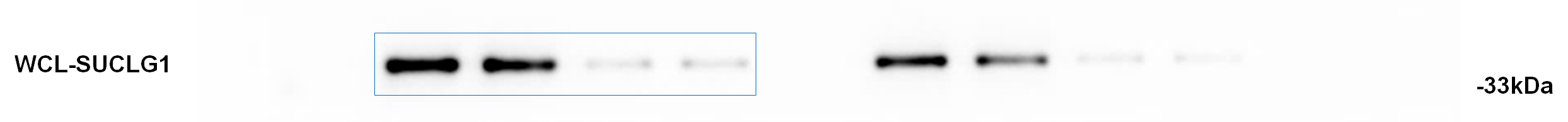

Supplement: Supplementary file 5 — Source data Fig. 4 [file 44318_2024_101_MOESM5_ESM.zip › Figure 4/4L/western WCL-SUCLG1.tif]

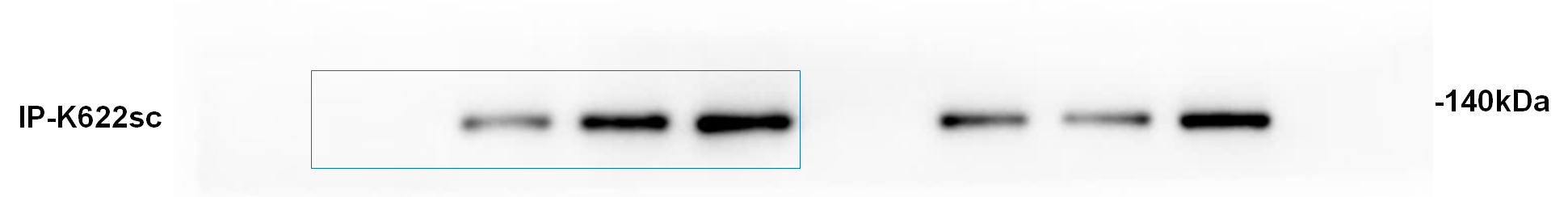

Supplement: Supplementary file 5 — Source data Fig. 4 [file 44318_2024_101_MOESM5_ESM.zip › Figure 4/4L/western IP-K622sc.tif]

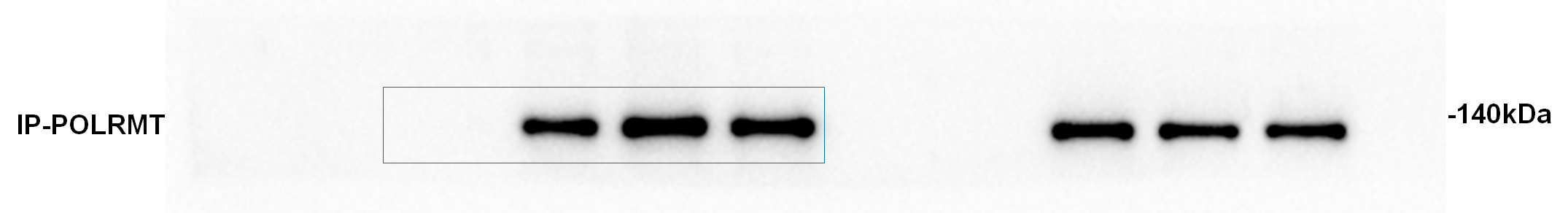

Supplement: Supplementary file 5 — Source data Fig. 4 [file 44318_2024_101_MOESM5_ESM.zip › Figure 4/4L/western IP-POLRMT.tif]

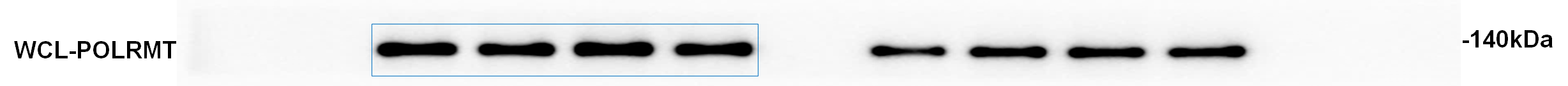

Supplement: Supplementary file 5 — Source data Fig. 4 [file 44318_2024_101_MOESM5_ESM.zip › Figure 4/4L/western WCL-POLRMT.tif]

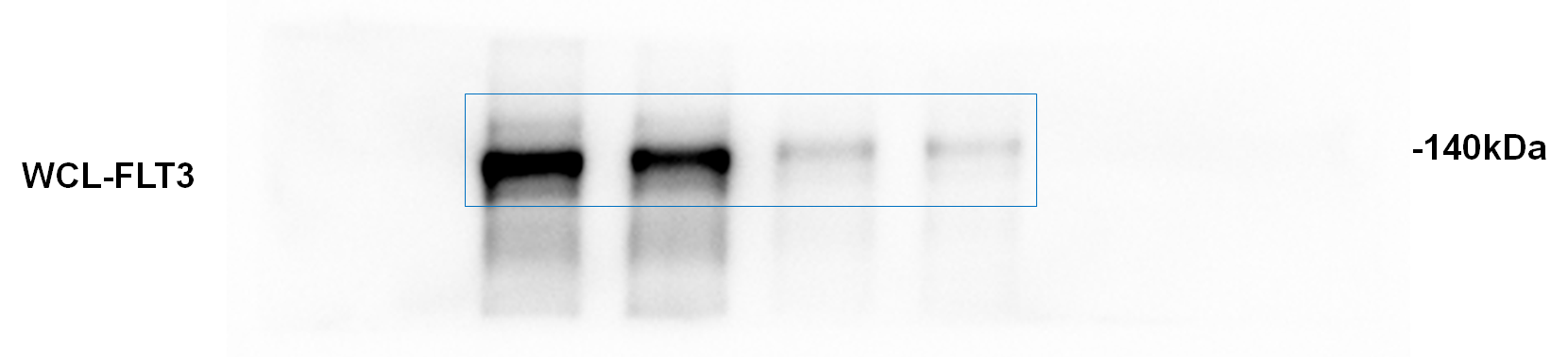

Supplement: Supplementary file 5 — Source data Fig. 4 [file 44318_2024_101_MOESM5_ESM.zip › Figure 4/4L/western WCL-FLT3.tif]

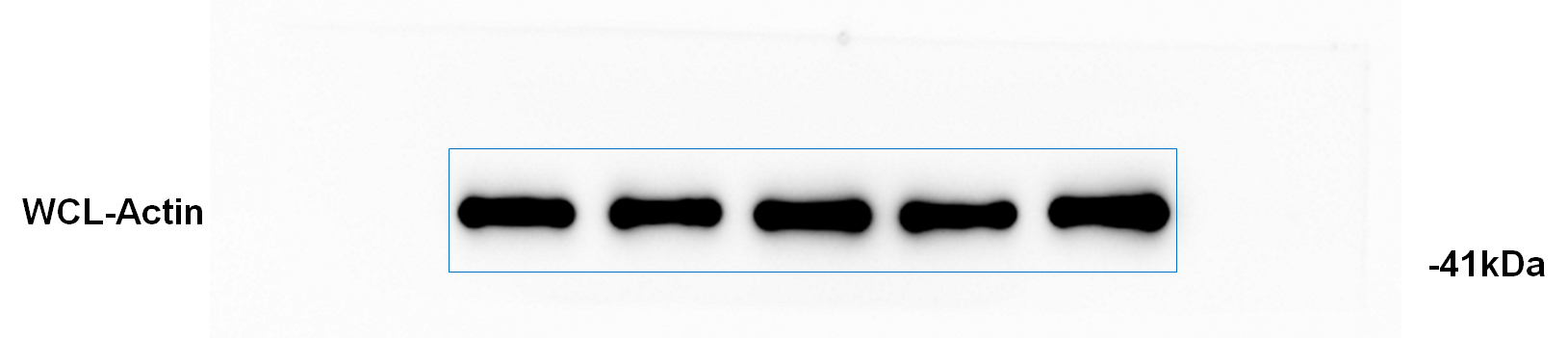

Supplement: Supplementary file 5 — Source data Fig. 4 [file 44318_2024_101_MOESM5_ESM.zip › Figure 4/4D/western WCL-Actin.tif]

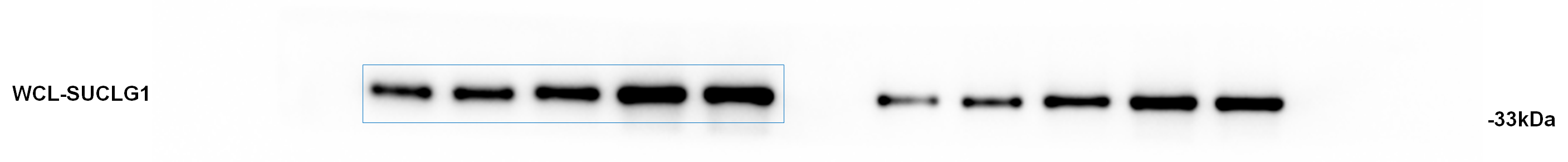

Supplement: Supplementary file 5 — Source data Fig. 4 [file 44318_2024_101_MOESM5_ESM.zip › Figure 4/4D/western WCL-SUCLG1.tif]

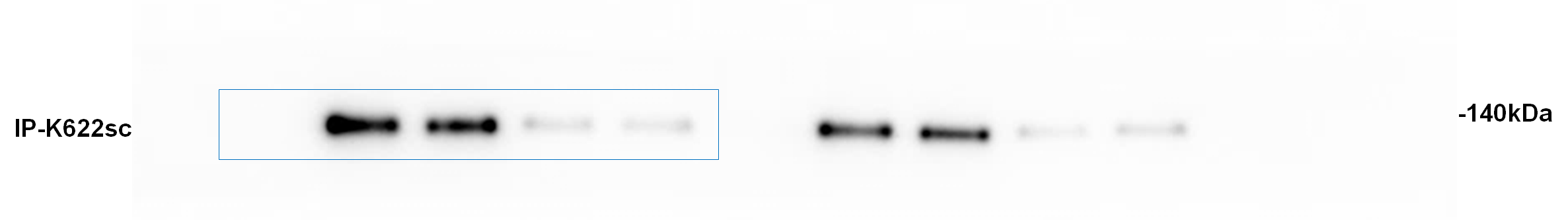

Supplement: Supplementary file 5 — Source data Fig. 4 [file 44318_2024_101_MOESM5_ESM.zip › Figure 4/4D/western IP-K622sc.tif]

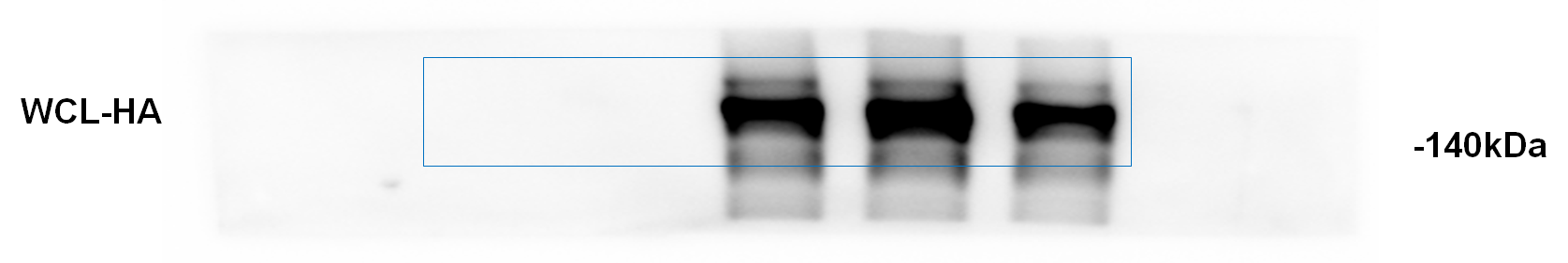

Supplement: Supplementary file 5 — Source data Fig. 4 [file 44318_2024_101_MOESM5_ESM.zip › Figure 4/4D/western WCL-HA.tif]

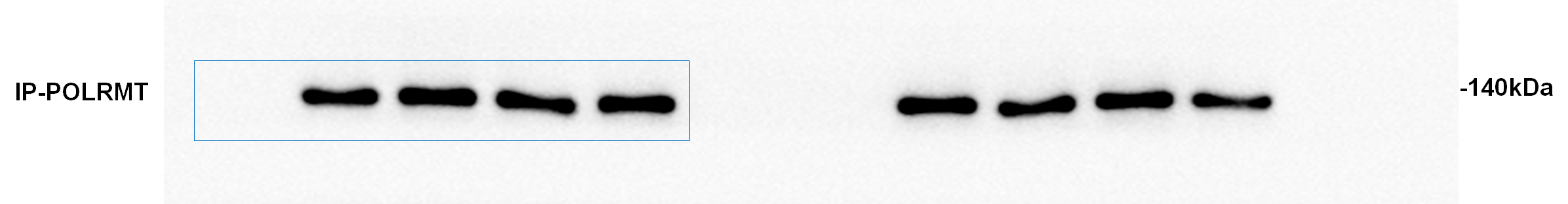

Supplement: Supplementary file 5 — Source data Fig. 4 [file 44318_2024_101_MOESM5_ESM.zip › Figure 4/4D/western IP-POLRMT.tif]

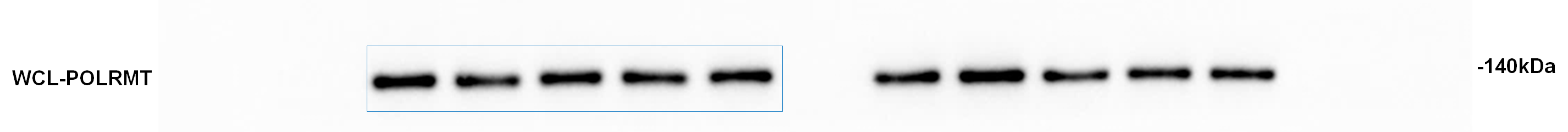

Supplement: Supplementary file 5 — Source data Fig. 4 [file 44318_2024_101_MOESM5_ESM.zip › Figure 4/4D/western WCL-POLRMT.tif]

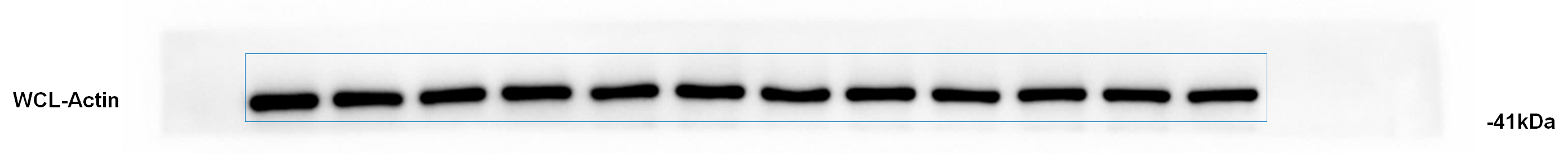

Supplement: Supplementary file 5 — Source data Fig. 4 [file 44318_2024_101_MOESM5_ESM.zip › Figure 4/4A/western WCL-Actin.tif]

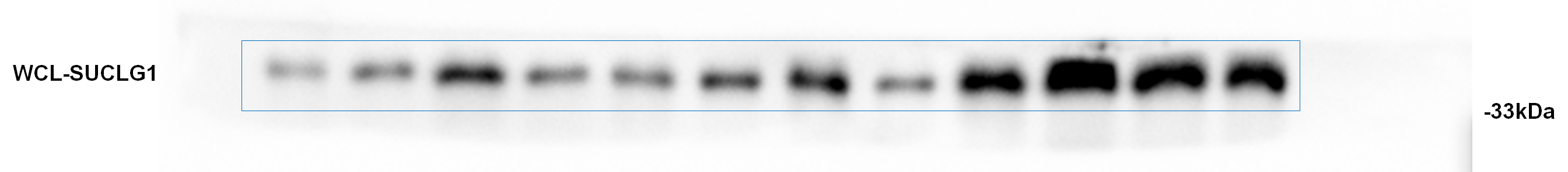

Supplement: Supplementary file 5 — Source data Fig. 4 [file 44318_2024_101_MOESM5_ESM.zip › Figure 4/4A/western WCL-SUCLG1.tif]

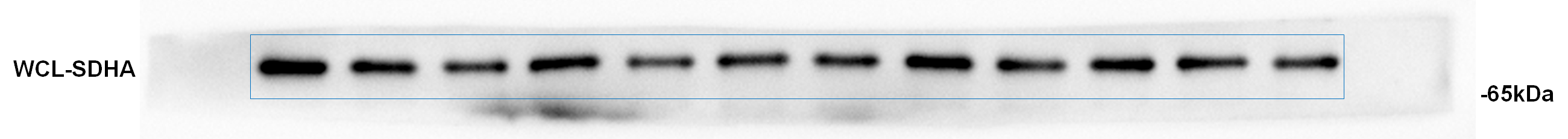

Supplement: Supplementary file 5 — Source data Fig. 4 [file 44318_2024_101_MOESM5_ESM.zip › Figure 4/4A/western WCL-SDHA.tif]

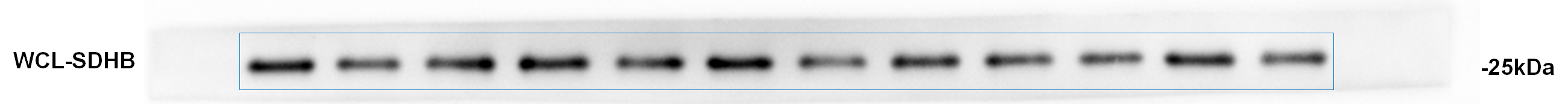

Supplement: Supplementary file 5 — Source data Fig. 4 [file 44318_2024_101_MOESM5_ESM.zip › Figure 4/4A/western WCL-SDHB.tif]

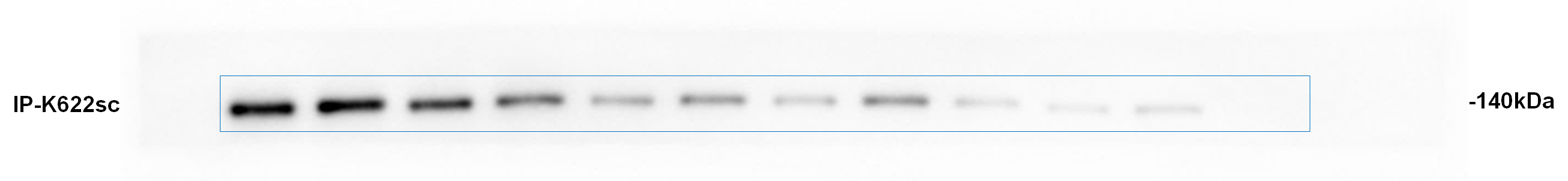

Supplement: Supplementary file 5 — Source data Fig. 4 [file 44318_2024_101_MOESM5_ESM.zip › Figure 4/4A/western IP-K622sc.tif]

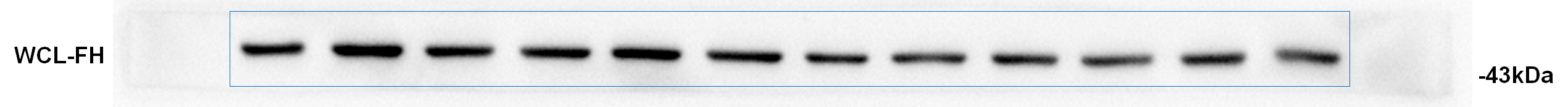

Supplement: Supplementary file 5 — Source data Fig. 4 [file 44318_2024_101_MOESM5_ESM.zip › Figure 4/4A/western WCL-FH.tif]

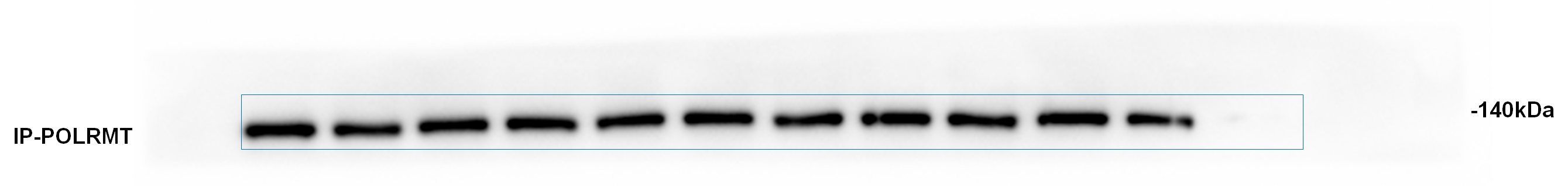

Supplement: Supplementary file 5 — Source data Fig. 4 [file 44318_2024_101_MOESM5_ESM.zip › Figure 4/4A/western IP-POLRMT.tif]

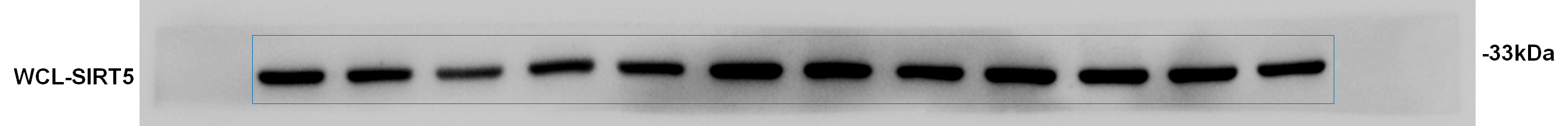

Supplement: Supplementary file 5 — Source data Fig. 4 [file 44318_2024_101_MOESM5_ESM.zip › Figure 4/4A/western WCL-SIRT5.tif]

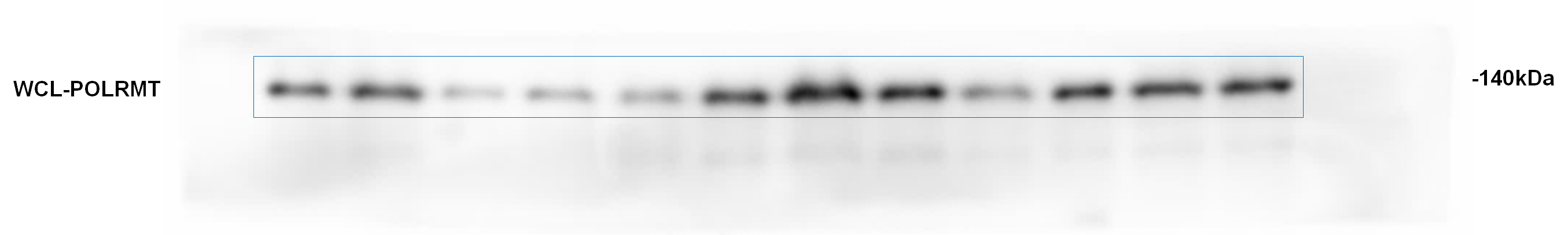

Supplement: Supplementary file 5 — Source data Fig. 4 [file 44318_2024_101_MOESM5_ESM.zip › Figure 4/4A/western WCL-POLRMT.tif]

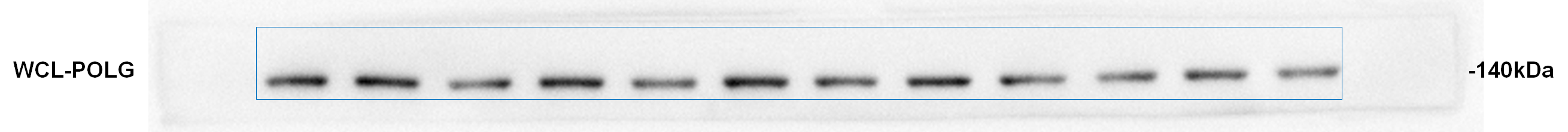

Supplement: Supplementary file 5 — Source data Fig. 4 [file 44318_2024_101_MOESM5_ESM.zip › Figure 4/4A/western WCL-POLG.tif]

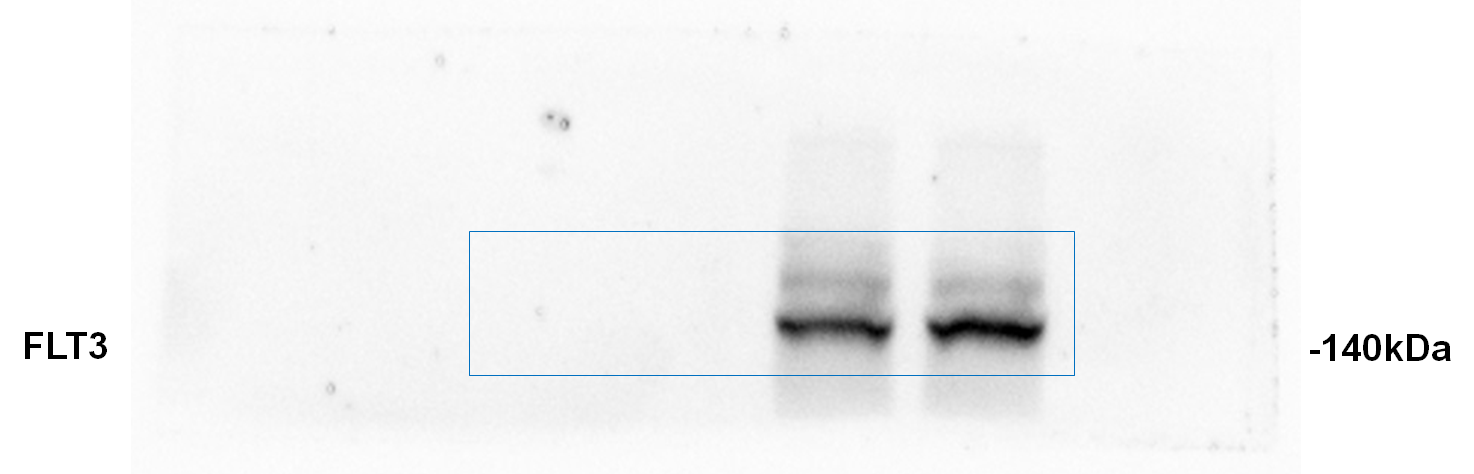

Supplement: Supplementary file 5 — Source data Fig. 4 [file 44318_2024_101_MOESM5_ESM.zip › Figure 4/4I/western FLT3.tif]
