# Supplementary figures and images for: SUCLG1 restricts POLRMT succinylation to enhance mitochondrial biogenesis and leukemia progression (part 2 of 2)
Source: EMBO J. 2024 Apr 22;43(12):2337–67. doi: 10.1038/s44318-024-00101-9 (PMC11183053; doi:10.1038/s44318-024-00101-9)

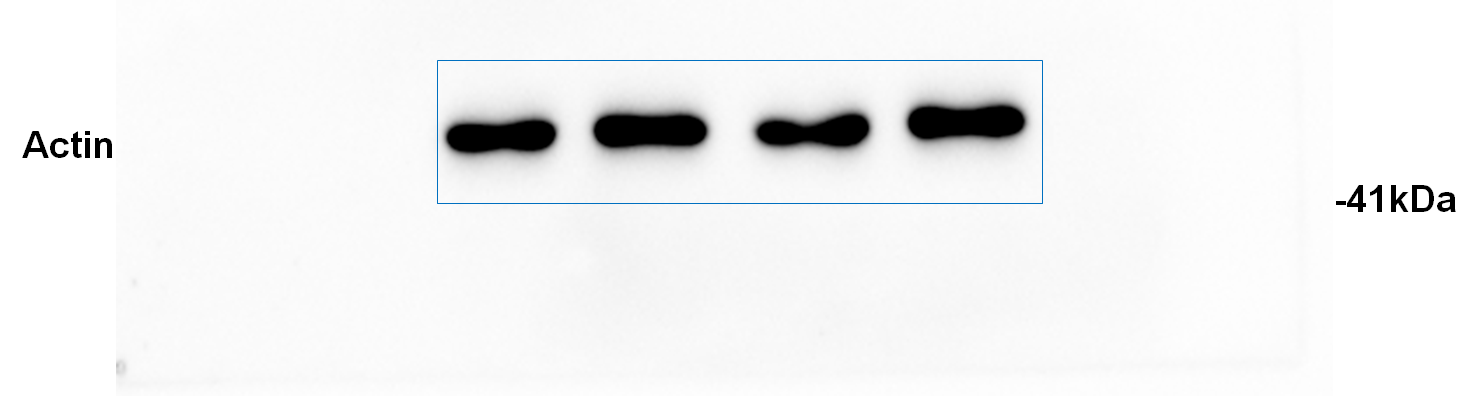

Supplement: Supplementary file 5 — Source data Fig. 4 [file 44318_2024_101_MOESM5_ESM.zip › Figure 4/4I/western Actin.tif]

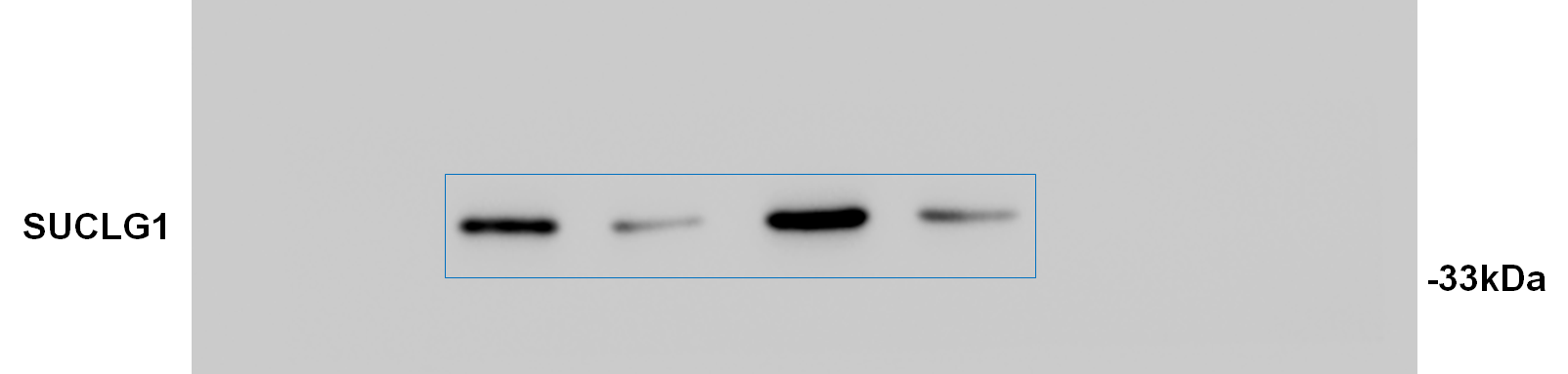

Supplement: Supplementary file 5 — Source data Fig. 4 [file 44318_2024_101_MOESM5_ESM.zip › Figure 4/4I/western SUCLG1.tif]

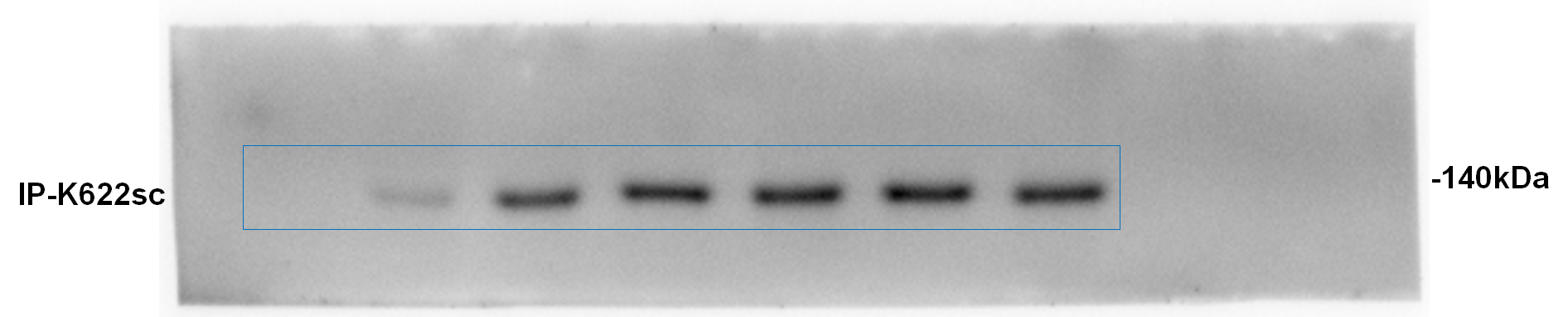

Supplement: Supplementary file 6 — Source data Fig. 5 [file 44318_2024_101_MOESM6_ESM.zip › Figure 5/5I/IP-K622sc.tif]

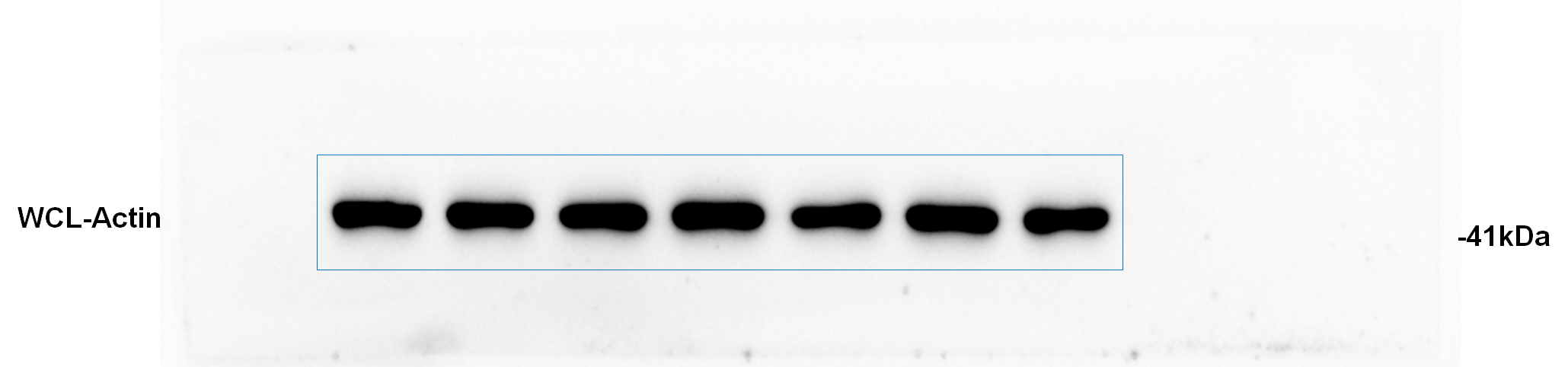

Supplement: Supplementary file 6 — Source data Fig. 5 [file 44318_2024_101_MOESM6_ESM.zip › Figure 5/5I/WCL-Actin.tif]

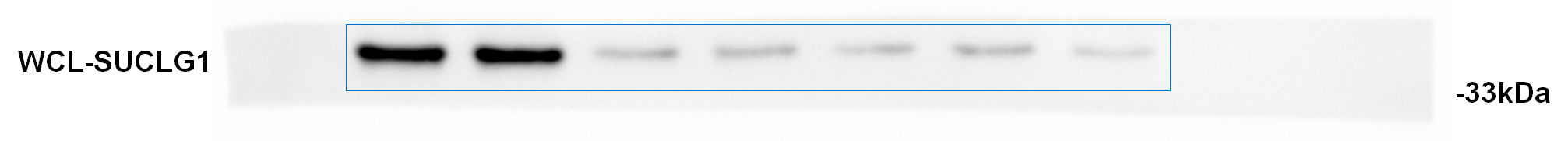

Supplement: Supplementary file 6 — Source data Fig. 5 [file 44318_2024_101_MOESM6_ESM.zip › Figure 5/5I/WCL-SUCLG1.tif]

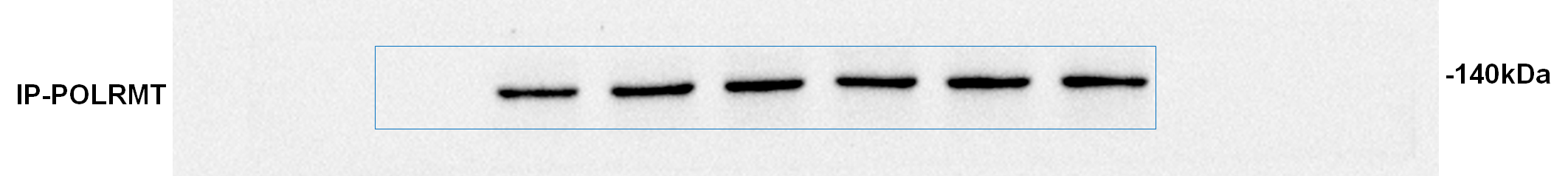

Supplement: Supplementary file 6 — Source data Fig. 5 [file 44318_2024_101_MOESM6_ESM.zip › Figure 5/5I/IP-POLRMT.tif]

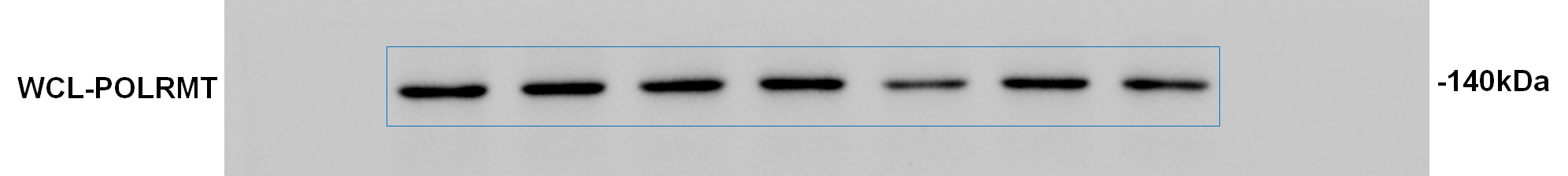

Supplement: Supplementary file 6 — Source data Fig. 5 [file 44318_2024_101_MOESM6_ESM.zip › Figure 5/5I/WCL-POLRMT.tif]

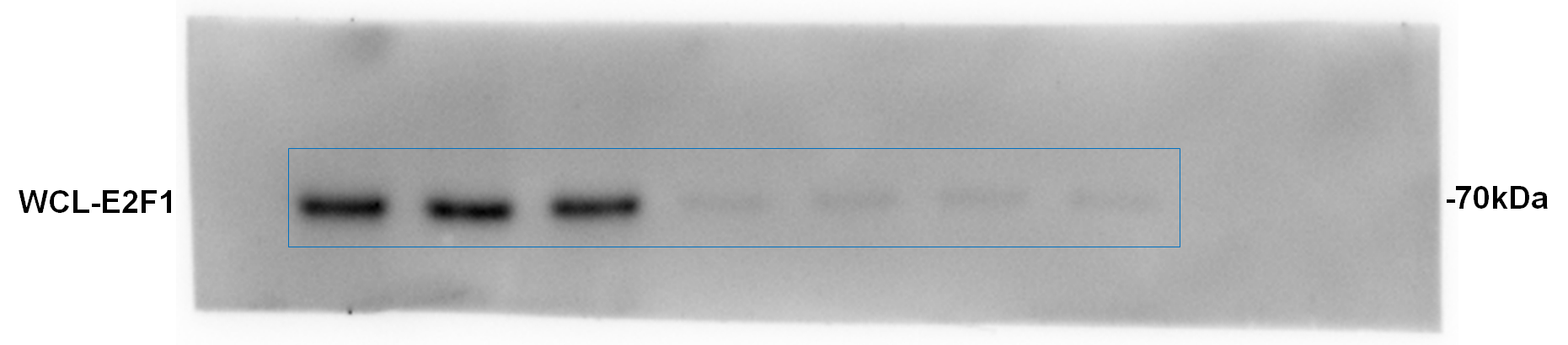

Supplement: Supplementary file 6 — Source data Fig. 5 [file 44318_2024_101_MOESM6_ESM.zip › Figure 5/5I/WCL-E2F1.tif]

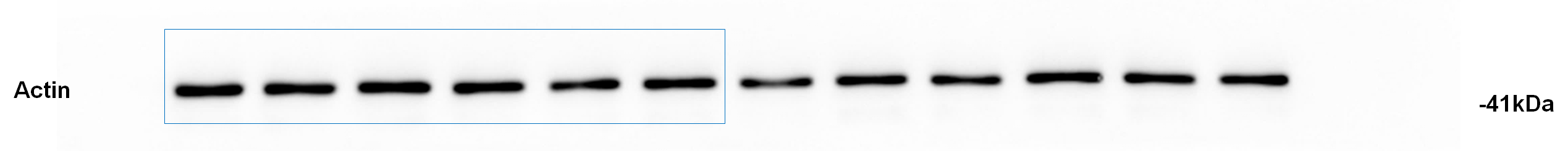

Supplement: Supplementary file 7 — Source data Fig. 6 [file 44318_2024_101_MOESM7_ESM.zip › Figure 6/6H/Actin.tif]

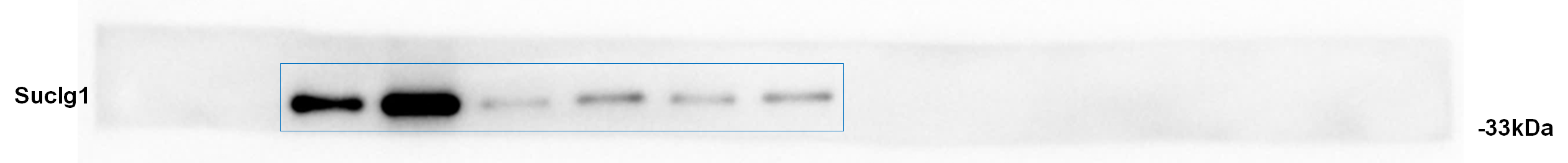

Supplement: Supplementary file 7 — Source data Fig. 6 [file 44318_2024_101_MOESM7_ESM.zip › Figure 6/6H/Suclg1.tif]

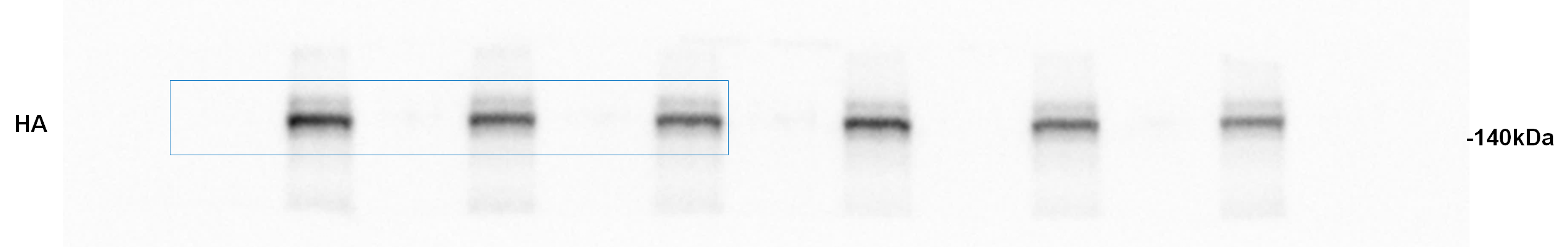

Supplement: Supplementary file 7 — Source data Fig. 6 [file 44318_2024_101_MOESM7_ESM.zip › Figure 6/6H/HA.tif]
