# Supplementary material for: SUCLG1 restricts POLRMT succinylation to enhance mitochondrial biogenesis and leukemia progression
Source: EMBO J. 2024 Apr 22;43(12):2337–67. doi: 10.1038/s44318-024-00101-9 (PMC11183053; doi:10.1038/s44318-024-00101-9)
Supplement: Supplementary file 10 — Expanded View Figures [file 44318_2024_101_MOESM10_ESM.pdf]

Expanded View Figures

Figure EV1. SUCLG1 maintains mtDNA-encoded gene expression and mitochondrial mass.

(**A**) Schematic overview of TCA cycle and the reaction catalyzed by SUCLG1. (**B**) Gene set enrichment analysis of the relation between *SUCLG1* and ETC, also known as oxidative phosphorylation, in TCGA AML and two other AML transcriptomic studies (GSE227839 and GSE185824), Kolmogorov–Smirnov test. (**C**) The top ten mitochondrial metabolic genes that positively correlate with ETC genes in TCGA AML. Shown are Spearman's correlation coefficients. (**D**) Scrambled control or shRNAs against *SUCLG1* were transduced into human $CD34^{+}$ CB cells or cancer cell lines as indicated. Total DNA was extracted to quantify mtDNA. Data represent means ± SD, $n = 3$ independent biological replicates, *t* test. $^{**}P < 0.01$. (**E**) Mitochondria were isolated from control or *SUCLG1*-knockdown MV411 cells. Whole-cell lysate and mitochondrial fractions were subject to western blotting. Histone H3, G6PD and HSP60 serve as markers for nucleus, cytosol and mitochondria, respectively. (**F**) Shown is the correlation between *SUCLG1* and ETC-related genes in various TCGA cancers. (**G**, **H**) mRNA expression of ETC-correlated genes was determined in control and *SUCLG1*-knockdown MV411 or HL60 cells. Data represent means ± SD, $n = 3$ independent biological replicates, *t* test. $^{**}P < 0.01$; $^{*}P < 0.05$; n.s. not significant. (**I**, **J**) Control and *SUCLG1*-knockdown MV411 cells were stained with CellROX Green (**I**). Cellular ROS levels were determined (**J**). Data represent means ± SD, $n = 3$ independent biological replicates, *t* test. $^{**}P < 0.01$. (**K**, **L**) Apoptosis of control and *SUCLG1*-knockdown MV411 cells was determined. Data represent means ± SD, $n = 3$ independent biological replicates, *t* test. $^{**}P < 0.01$. (**M**) Stable MV411 cells were treated with 0.1 µM doxorubicin for 4 days, senescence was visualized by β-galactosidase staining. Scale bars: 50 µm. (**N**) BrdU incorporation was assayed in control and *SUCLG1*-knockdown MV411 cells. Data represent means ± SD, $n = 3$ independent biological replicates, *t* test. $^{**}P < 0.01$. (**O**) Distribution of stable MV411 cells in cell cycle was determined. Data represent means ± SD, $n = 3$ independent biological replicates, *t* test. $^{**}P < 0.01$; $^{*}P < 0.05$. (**P**) Multiple alignments of amino acid sequences adjacent to P170 and A209 of SUCLG1. (**Q**, **R**) The structural locations of P170 and A209 residues were analyzed (PDB ID: 6XRU). The catalytic center contains succinate, Mg^{2+}, and desulfo-CoA (succinyl-CoA analog). (**S**) Flag-tagged SUCLG1 and disease-derived mutants (P170R and A209E) were expressed in HEK293T cells. SUCLG1 enzymes were immunopurified and subjected to catalytic activity assay. Enzyme activities were normalized to Flag-tagged protein. Data represent means ± SD, $n = 3$ independent biological replicates, *t* test. $^{**}P < 0.01$. Source data are available online for this figure.

 © The Author(s)

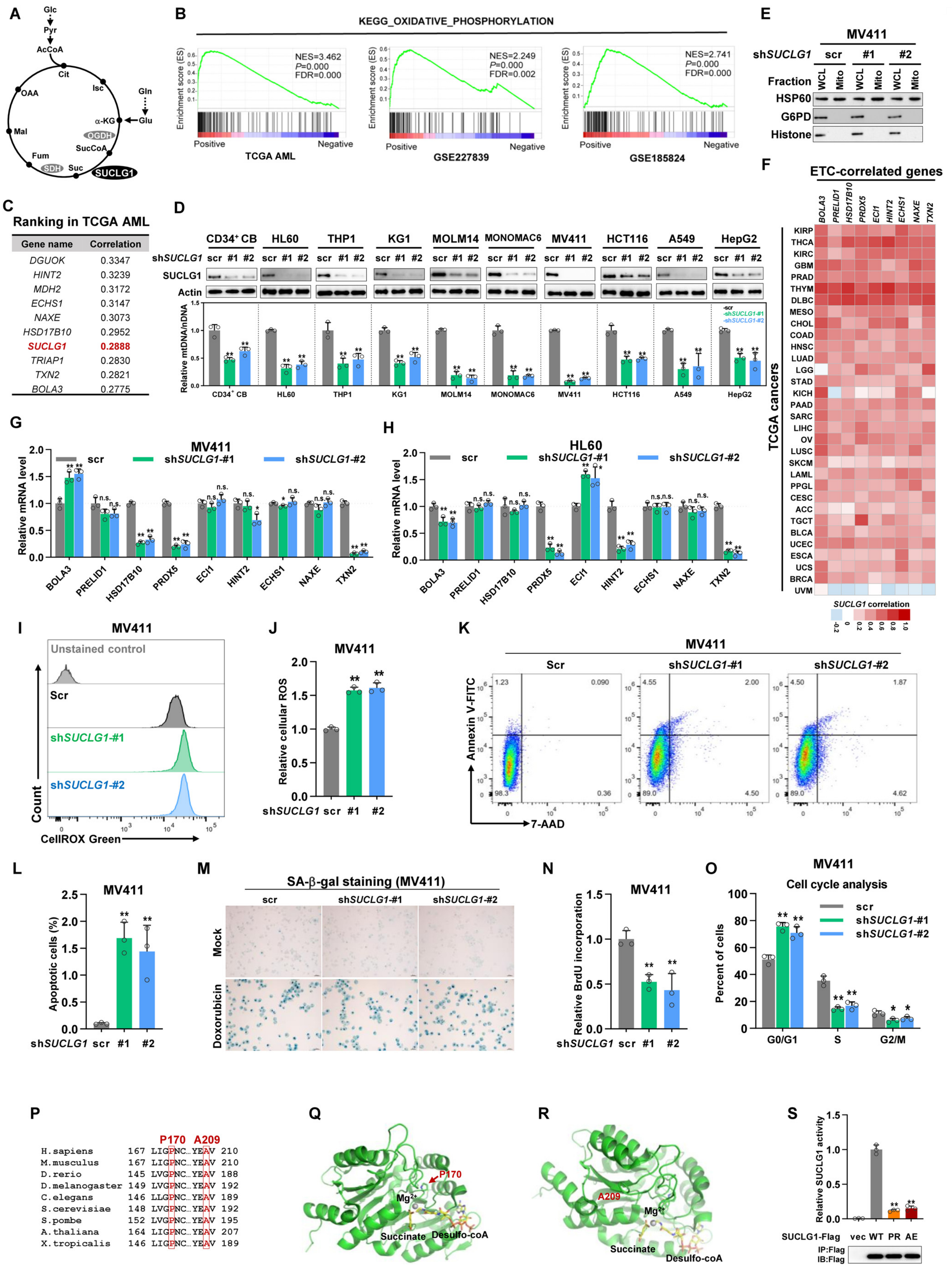
A
Glc
Pyr
AcCoA
Cit
Isc
OAA
Gln
α-KG
Glu
Mal
OGDH
SucCoA
Fum
SDH
Suc
SUCLG1
B
KEGG_OXIDATIVE_PHOSPHORYLATION
NES=3.462
P=0.000
FDR=0.000
TCGA AML
NES=2.249
P=0.000
FDR=0.002
GSE227839
NES=2.741
P=0.000
FDR=0.000
GSE185824
Enrichment score (ES)
Positive
Negative
C
Ranking in TCGA AML
Gene name Correlation
DGUOK 0.3347
HINT2 0.3239
MDH2 0.3172
ECHS1 0.3147
NAXE 0.3073
HSD17B10 0.2952
SUCLG1 0.2888
TRIAP1 0.2830
TXN2 0.2821
BOLA3 0.2775
D
CD34+ CB
HL60
THP1
KG1
MOLM14
MONOMAC6
MV411
HCT116
A549
HepG2
shSUCLG1 scr #1 #2
SUCLG1
Actin
Relative mtDNA/nDNA
E
MV411
shSUCLG1 scr #1 #2
Fraction WCL Mito
HSP60
G6PD
Histone
F
ETC-correlated genes
BOLA3 PRELID1 HSD17B10 PRDX5 ECI1 HINT2 ECHS1 NAXE TXN2
TCGA cancers
KIRP THCA KIRC GBM PRAD THYM DLBC MESO CHOL COAD HNSC LUAD LGG STAD KICH PAAD SARC LIHC OV LUSC SKCM LAML PPGL CESC ACC TGCT BLCA UCEC ESCA UCS BRCA UVM
SUCLG1 correlation
G
MV411
Relative mRNA level
scr shSUCLG1-#1 shSUCLG1-#2
BOLA3 PRELID1 HSD17B10 PRDX5 ECI1 HINT2 ECHS1 NAXE TXN2
H
HL60
I
MV411
Unstained control
Scr
shSUCLG1-#1
shSUCLG1-#2
Count
CellROX Green
J
MV411
Relative cellular ROS
shSUCLG1 scr #1 #2
K
MV411
Scr
shSUCLG1-#1
shSUCLG1-#2
Annexin V-FITC
7-AAD
L
MV411
Apoptotic cells (%)
shSUCLG1 scr #1 #2
M
SA-β-gal staining (MV411)
scr shSUCLG1-#1 shSUCLG1-#2
Mock
Doxorubicin
N
MV411
Relative BrdU incorporation
shSUCLG1 scr #1 #2
O
MV411
Cell cycle analysis
scr shSUCLG1-#1 shSUCLG1-#2
Percent of cells
G0/G1 S G2/M
P
P170 A209
H.sapiens 167 LIGPNC…YEAV 210
M.musculus 167 LIGPNC…YEAV 210
D.rerio 145 LVGPNC…YEAV 188
D.melanogaster 149 LVGPNC…YEAV 192
C.elegans 146 LLGPNC…YEAV 189
S.cerevisiae 148 LVGPNC…YEAV 192
S.pombe 152 LVGPNC…YEAV 195
A.thaliana 164 LIGPNC…YEAV 207
X.tropicalis 146 LIGPNC…YEAV 189
Q
P170
Mg2+
Succinate
Desulfo-coA
R
A209
Mg2+
Succinate
Desulfo-coA
S
Relative SUCLG1 activity
SUCLG1-Flag vec WT PR AE
IP:Flag
IB:Flag

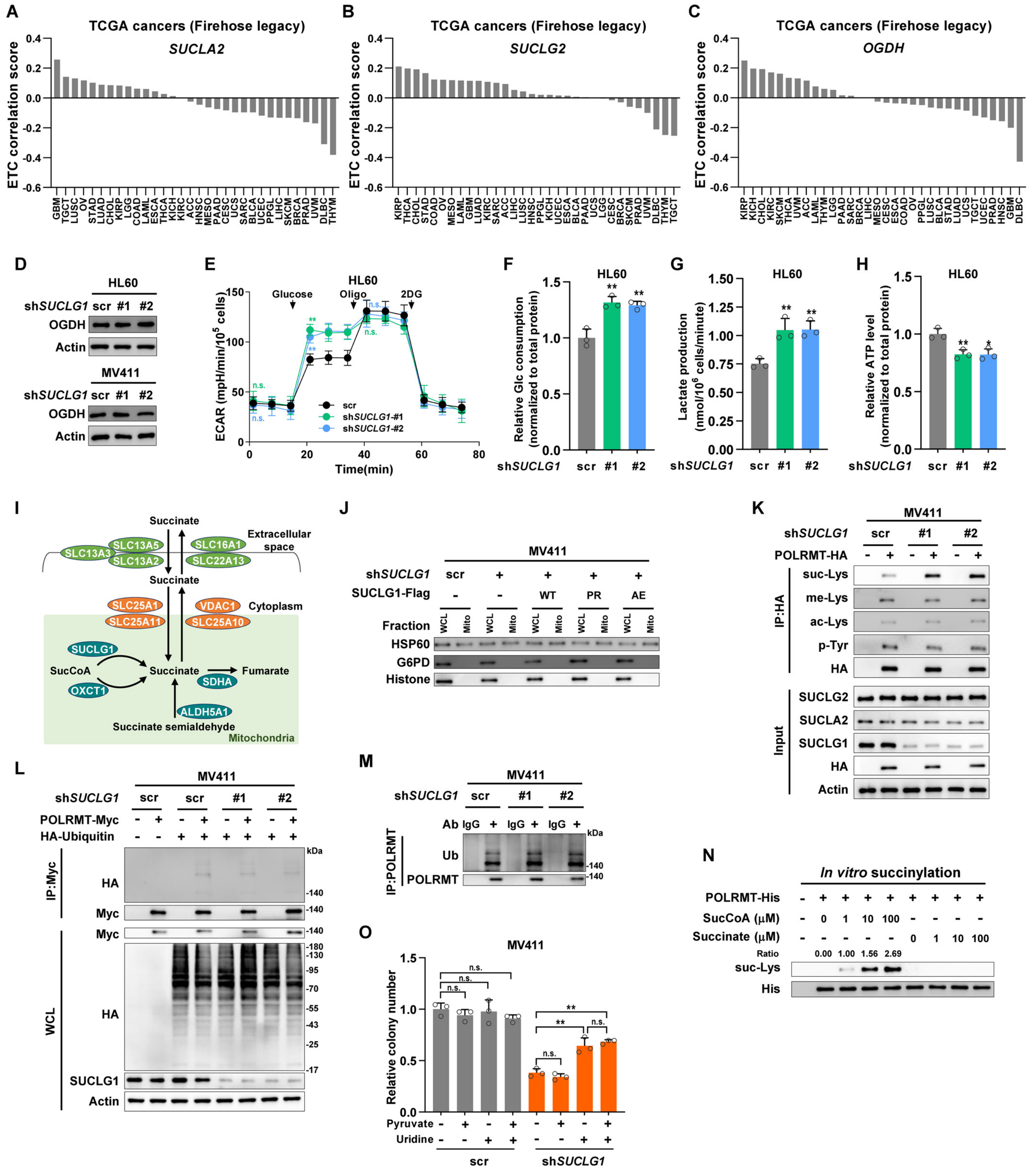
A
TCGA cancers (Firehose legacy)
SUCLA2
ETC correlation score
GBM TGCT LUSC OV STAD LUAD CHOL KIRP LGG COAD LAML ESCA THCA KICH KIRC ACC HNSC MESO PAAD CESC UCS SARC BLCA UCEC PPGL LIHC SKCM BRCA PRAD UVM DLBC THYM
B
TCGA cancers (Firehose legacy)
SUCLG2
ETC correlation score
KIRP THCA CHOL STAD COAD OV MESO LAML GBM LUAD KIRC SARC ACC LIHC LUSC HNSC PPGL KICH UCEC ESCA BLCA PAAD UCS LGG CESC BRCA SKCM PRAD UVM DLBC THYM TGCT
C
TCGA cancers (Firehose legacy)
OGDH
ETC correlation score
KIRP KICH CHOL KIRC SKCM THCA UVM ACC LAML THYM LGG PAAD SARC LIHC MESO CESC ESCA COAD OV PPGL LUSC BLCA STAD LUAD UCS TGCT PRAD HNSC GBM DLBC
D
HL60
shSUCLG1 scr #1 #2
OGDH
Actin
MV411
shSUCLG1 scr #1 #2
OGDH
Actin
E
HL60
Glucose
Oligo
2DG
ECAR (mpH/min/10^5 cells)
Time(min)
scr
shSUCLG1-#1
shSUCLG1-#2
F
HL60
Relative Glc consumption (normalized to total protein)
shSUCLG1 scr #1 #2
G
HL60
Lactate production (nmol/10^6 cells/minute)
shSUCLG1 scr #1 #2
H
HL60
Relative ATP level (normalized to total protein)
shSUCLG1 scr #1 #2
I
Succinate
Extracellular space
SLC13A3
SLC13A5
SLC13A2
SLC16A1
SLC22A13
Succinate
SLC25A1
SLC25A11
VDAC1
SLC25A10
Cytoplasm
SUCLG1
SucCoA
Succinate
Fumarate
SDHA
OXCT1
ALDH5A1
Succinate semialdehyde
Mitochondria
J
MV411
shSUCLG1 scr + + + +
SUCLG1-Flag - - WT PR AE
Fraction WCL Mito
HSP60
G6PD
Histone
K
MV411
shSUCLG1 scr #1 #2
POLRMT-HA
IP:HA
suc-Lys
me-Lys
ac-Lys
p-Tyr
HA
Input
SUCLG2
SUCLA2
SUCLG1
HA
Actin
L
MV411
shSUCLG1 scr scr #1 #2
POLRMT-Myc
HA-Ubiquitin
IP:Myc
HA
Myc
WCL
Myc
HA
SUCLG1
Actin
kDa
M
MV411
shSUCLG1 scr #1 #2
Ab IgG + IgG + IgG +
IP:POLRMT
Ub
POLRMT
kDa
N
In vitro succinylation
POLRMT-His
SucCoA (μM) 0 1 10 100
Succinate (μM) 0 1 10 100
Ratio 0.00 1.00 1.56 2.69
suc-Lys
His
O
MV411
Relative colony number
Pyruvate
Uridine
scr
shSUCLG1

© The Author(s)

Figure EV2. SUCLG1 reduces succinyl-CoA level to restrict POLRMT succinylation.

(**A–C**) ETC correlation scores of succinyl-CoA-metabolizing enzymes, *SUCLA2* (**A**), *SUCLG2* (**B**), and *OGDH* (**C**), were determined in various TCGA cancers. Shown are Spearman's correlation coefficients. (**D**) OGDH protein expression in stable HL60 and MV411 cells was determined by western blotting. (**E**) Extracellular acidification rates of control or *SUCLG1*-knockdown HL60 cells were determined. Data represent means ± SD, $n = 3$ independent biological replicates, *t* test. $^{**}P < 0.01$; n.s. not significant. (**F**, **G**) Glucose (Glc) consumption and lactate production were assayed in control and *SUCLG1*-knockdown HL60 cells. Data represent means ± SD, $n = 3$ independent biological replicates, *t* test. $^{**}P < 0.01$. (**H**) ATP abundance was quantified in control and *SUCLG1*-knockdown HL60 cells, and normalized to total protein. Data represent means ± SD, $n = 3$ independent biological replicates, *t* test. $^{**}P < 0.01$; $^{*}P < 0.05$. (**I**) Schematic overview of metabolic enzymes and transporters involved in succinate metabolism. (**J**) Mitochondria were isolated from stable *SUCLG1*-knockdown and rescue MV411 cells. Whole-cell lysate and mitochondrial fractions were subject to western blotting. Histone H3, G6PD, and HSP60 were included as markers for the nucleus, cytosol, and mitochondria, respectively. (**K**) HA-tagged POLRMT was expressed in scrambled control or *SUCLG1*-knockdown MV411 cells. POLRMT-HA was immunoprecipitated from stable MV411 cells and subjected to western blotting to determine lysine succinylation (suc-Lys), lysine methylation (me-Lys), lysine acetylation (ac-Lys), and tyrosine phosphorylation (p-Tyr). (**L**) Myc-tagged POLRMT and HA-tagged ubiquitin were co-expressed in stable MV411 cells. POLRMT was immunopurified with Myc antibody to determine its ubiquitination by western blotting. (**M**) Endogenous POLRMT was immunoprecipitated from control or *SUCLG1*-knockdown MV411 cells. Ubiquitination was determined by western blotting. (**N**) Bacterially expressed His-tagged POLRMT was purified and incubated with succinyl-CoA (sucCoA) or succinate in vitro. Lysine succinylation levels were determined by western blotting and normalized to POLRMT-His protein (ratio). (**O**) Scrambled control or *SUCLG1*-knockdown (sh-#1) MV411 cells were cultured with 1 mM pyruvate or 0.2 mM uridine. Colony formation was determined. Data represent means ± SD, $n = 3$ independent biological replicates, *t* test. $^{**}P < 0.01$; n.s. not significant. Source data are available online for this figure.

© The Author(s)

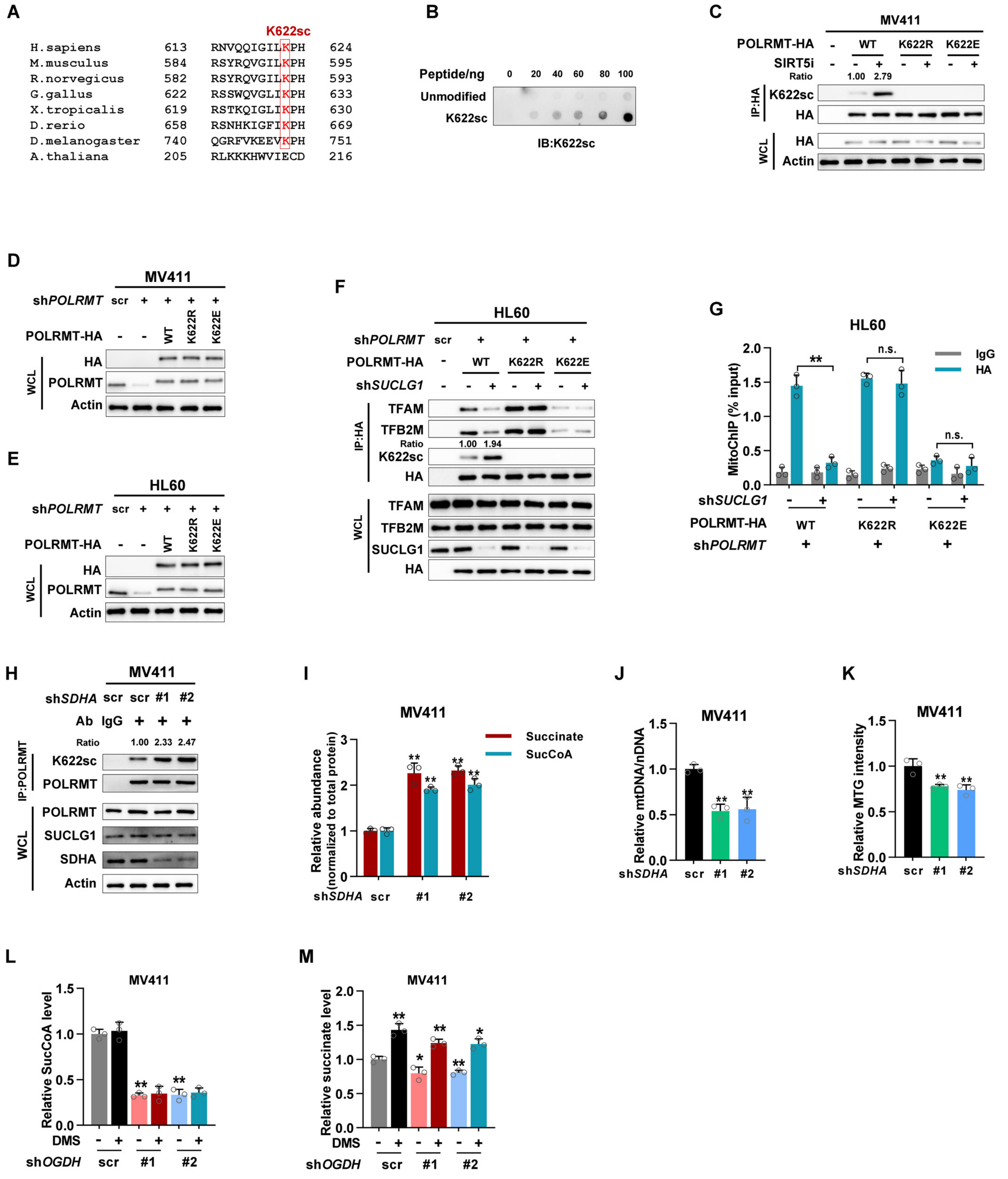
A
K622sc
H.sapiens 613 RNVQQIGILKPH 624
M.musculus 584 RSYRQVGILKPH 595
R.norvegicus 582 RSYRQVGILKPH 593
G.gallus 622 RSSWQVGLIKPH 633
X.tropicalis 619 RSTKQIGLIKPH 630
D.rerio 658 RSNHKIGFIKPH 669
D.melanogaster 740 QGRFVKEEVKPH 751
A.thaliana 205 RLKKKHWVIECD 216
B
Peptide/ng 0 20 40 60 80 100
Unmodified
K622sc
IB:K622sc
C
MV411
POLRMT-HA - WT K622R K622E
SIRT5i - - + - + - +
Ratio 1.00 2.79
IP:HA K622sc
HA
WCL HA
Actin
D
MV411
shPOLRMT scr + + + +
POLRMT-HA - - WT K622R K622E
WCL HA
POLRMT
Actin
E
HL60
shPOLRMT scr + + + +
POLRMT-HA - - WT K622R K622E
WCL HA
POLRMT
Actin
F
HL60
shPOLRMT scr + + +
POLRMT-HA - WT K622R K622E
shSUCLG1 - - + - + - +
IP:HA TFAM
TFB2M
Ratio 1.00 1.94
K622sc
HA
WCL TFAM
TFB2M
SUCLG1
HA
G
HL60
MitoChIP (% input)
IgG
HA
**
n.s.
n.s.
shSUCLG1 - + - + - +
POLRMT-HA WT K622R K622E
shPOLRMT + + +
H
MV411
shSDHA scr scr #1 #2
Ab IgG + + +
Ratio 1.00 2.33 2.47
IP:POLRMT K622sc
POLRMT
WCL POLRMT
SUCLG1
SDHA
Actin
I
MV411
Succinate
SucCoA
Relative abundance (normalized to total protein)
shSDHA scr #1 #2
J
MV411
Relative mtDNA/nDNA
shSDHA scr #1 #2
K
MV411
Relative MTG intensity
shSDHA scr #1 #2
L
MV411
Relative SucCoA level
DMS - + - + - +
shOGDH scr #1 #2
M
MV411
Relative succinate level
DMS - + - + - +
shOGDH scr #1 #2

 © The Author(s)

Figure EV3. K622 succinylation suppresses POLRMT activity.

(**A**) Multiple alignments of amino acid sequences corresponding to K622 of POLRMT from various model organisms. (**B**) Dot blotting was performed to test the specificity of K622 succinylation antibody. Unmodified peptide was included as the negative control. (**C**) HA-tagged POLRMT and its K622 mutants were expressed in MV411 cells. Cells were treated with SIRT5 inhibitor (SIRT5i). POLRMT was immunopurified with HA antibody to determine K622 succinylation. K622sc signal was normalized to POLRMT-HA protein (ratio). (**D**, **E**) HA-tagged POLRMT and its K622 mutants were re-expressed in *POLRMT*-knockdown MV411 and HL60 cells at physiologically relevant levels. (**F**, **G**) Control or shRNA against *SUCLG1* was expressed in *POLRMT*-knockdown and rescue HL60 cells. POLRMT-HA was immunopurified to assay protein interaction and K622 succinylation. K622sc signal was normalized to POLRMT-HA protein (ratio) (**F**). MitoChIP was performed to determine mtDNA binding of POLRMT-HA (**G**). Data represent means ± SD, $n = 3$ independent biological replicates, *t* test. **$P < 0.01$; n.s. not significant. (**H**) Endogenous POLRMT was immunoprecipitated from control or *SDHA*-knockdown MV411 cells. K622 succinylation was determined by western blotting. (**I**) Metabolites were extracted from control and *SDHA*-knockdown MV411 cells. Succinate and succinyl-CoA levels were quantified by mass spectrometry and normalized to total protein. Data represent means ± SD, $n = 3$ independent biological replicates, *t* test. **$P < 0.01$. (**J**, **K**) mtDNA abundance and mitochondrial mass were determined by qPCR and MTG staining, respectively. Data represent means ± SD, $n = 3$ independent biological replicates, *t* test. **$P < 0.01$. (**L**, **M**) Scrambled control or *OGDH*-knockdown stable MV411 cells were cultured with or without 50 μM dimethyl succinate (DMS). Succinyl-CoA and succinate levels were quantified by mass spectrometry and normalized to total protein. Data represent means ± SD, $n = 3$ independent biological replicates, *t* test. **$P < 0.01$; *$P < 0.05$. Source data are available online for this figure.

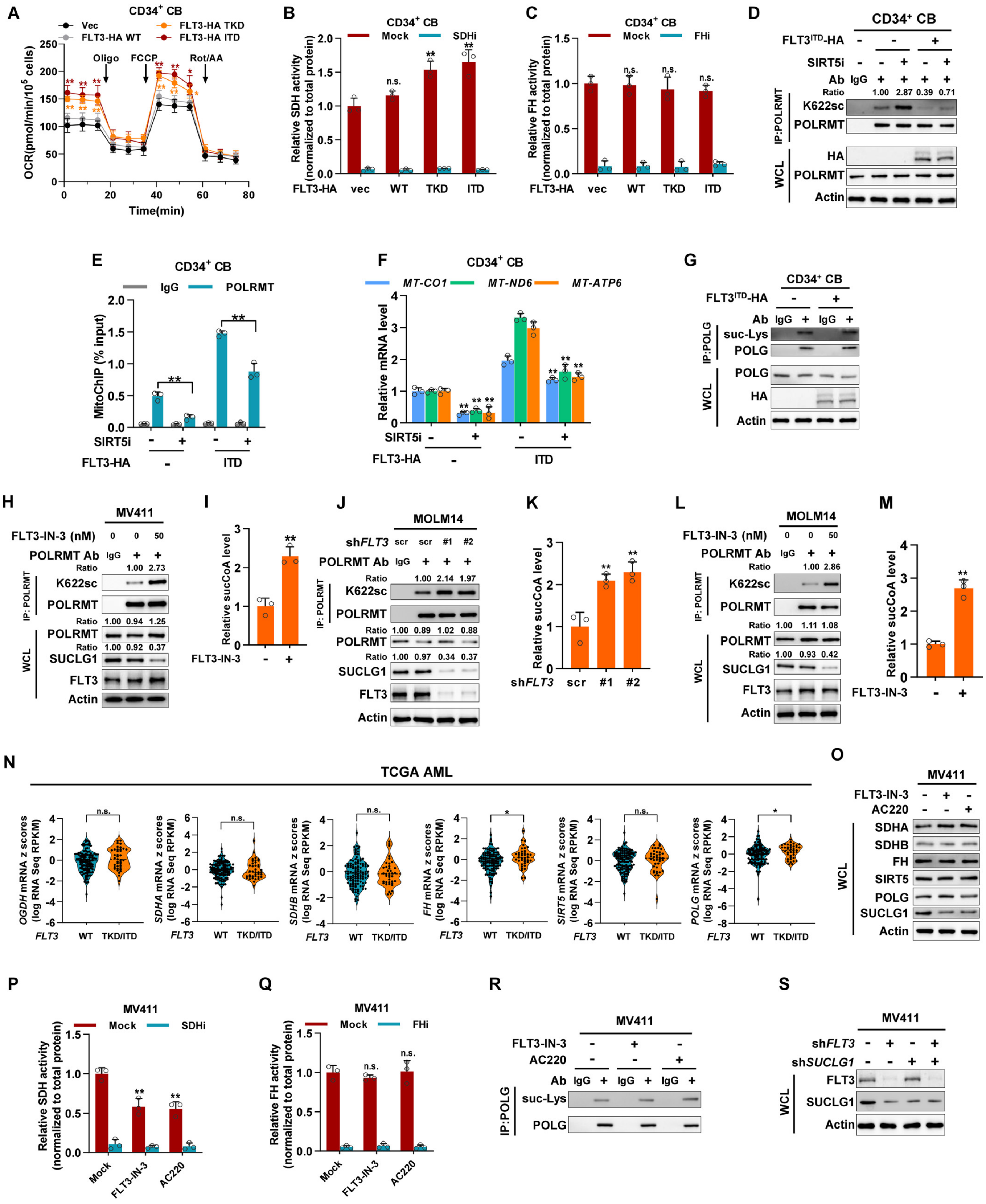
A
CD34+ CB
Vec
FLT3-HA WT
FLT3-HA TKD
FLT3-HA ITD
Oligo
FCCP
Rot/AA
OCR(pmol/min/10^5 cells)
Time(min)
B
CD34+ CB
Mock
SDHi
Relative SDH activity (normalized to total protein)
FLT3-HA vec WT TKD ITD
C
CD34+ CB
Mock
FHi
Relative FH activity (normalized to total protein)
FLT3-HA vec WT TKD ITD
D
CD34+ CB
FLT3ITD-HA
SIRT5i
Ab IgG
Ratio 1.00 2.87 0.39 0.71
IP:POLRMT
K622sc
POLRMT
WCL
HA
POLRMT
Actin
E
CD34+ CB
IgG
POLRMT
MitoChIP (% input)
SIRT5i
FLT3-HA - ITD
F
CD34+ CB
MT-CO1
MT-ND6
MT-ATP6
Relative mRNA level
SIRT5i
FLT3-HA - ITD
G
CD34+ CB
FLT3ITD-HA
Ab IgG + IgG +
IP:POLG
suc-Lys
POLG
WCL
POLG
HA
Actin
H
MV411
FLT3-IN-3 (nM) 0 0 50
POLRMT Ab IgG + +
Ratio 1.00 2.73
IP: POLRMT
K622sc
POLRMT
Ratio 1.00 0.94 1.25
WCL
POLRMT
Ratio 1.00 0.92 0.37
SUCLG1
FLT3
Actin
I
Relative sucCoA level
FLT3-IN-3 - +
J
MOLM14
shFLT3 scr scr #1 #2
POLRMT Ab IgG + + +
Ratio 1.00 2.14 1.97
IP: POLRMT
K622sc
POLRMT
Ratio 1.00 0.89 1.02 0.88
POLRMT
Ratio 1.00 0.97 0.34 0.37
SUCLG1
FLT3
Actin
K
Relative sucCoA level
shFLT3 scr #1 #2
L
MOLM14
FLT3-IN-3 (nM) 0 0 50
POLRMT Ab IgG + +
Ratio 1.00 2.86
IP: POLRMT
K622sc
POLRMT
Ratio 1.00 1.11 1.08
WCL
POLRMT
Ratio 1.00 0.93 0.42
SUCLG1
FLT3
Actin
M
Relative sucCoA level
FLT3-IN-3 - +
N
TCGA AML
OGDH mRNA z scores (log RNA Seq RPKM)
SDHA mRNA z scores (log RNA Seq RPKM)
SDHB mRNA z scores (log RNA Seq RPKM)
FH mRNA z scores (log RNA Seq RPKM)
SIRT5 mRNA z scores (log RNA Seq RPKM)
POLG mRNA z scores (log RNA Seq RPKM)
FLT3 WT TKD/ITD
O
MV411
FLT3-IN-3
AC220
WCL
SDHA
SDHB
FH
SIRT5
POLG
SUCLG1
Actin
P
MV411
Mock
SDHi
Relative SDH activity (normalized to total protein)
Mock FLT3-IN-3 AC220
Q
MV411
Mock
FHi
Relative FH activity (normalized to total protein)
Mock FLT3-IN-3 AC220
R
MV411
FLT3-IN-3
AC220
Ab IgG + IgG + IgG +
IP:POLG
suc-Lys
POLG
S
MV411
shFLT3
shSUCLG1
WCL
FLT3
SUCLG1
Actin

 © The Author(s)

Figure EV4. Leukemia-derived FLT3 mutants upregulate SUCLG1 to boost mitobiogenesis.

(**A**) HA-tagged FLT3 and its mutants were stably expressed in $CD34^+$ CB cells. Oxygen consumption rates were determined by Seahorse experiments. Data represent means ± SD, $n = 3$ independent biological replicates, *t* test. $^{**}P < 0.01$; $^{*}P < 0.05$. (**B**, **C**) Enzymatic activities of SDH and FH in stable $CD34^+$ CB cells were determined and normalized to total protein. Data represent means ± SD, $n = 3$ independent biological replicates, *t* test. $^{**}P < 0.01$; n.s. not significant. (**D–F**) Stable $CD34^+$ CB cells were treated with or without SIRT5 inhibitor. K622 succinylation (**D**), mtDNA binding (**E**), and mtDNA-encoded gene expression (**F**) were determined. Data represent means ± SD, $n = 3$ independent biological replicates, *t* test. $^{**}P < 0.01$. (**G**) Endogenous POLG was immunoprecipitated from control or $FLT3^{ITD}$-expressing $CD34^+$ CB cells. Lysine succinylation was determined by western blotting. (**H**, **I**) MV411 cells were treated with FLT3-IN-3, a chemical inhibitor of FLT3, for 24 h. Endogenous POLRMT was immunopurified to determine K622 succinylation. K622sc levels were normalized to POLRMT protein (ratio) (**H**). Cellular succinyl-CoA levels were determined and normalized to total protein (**I**). Data represent means ± SD, $n = 3$ independent biological replicates, *t* test. $^{**}P < 0.01$. (**J**, **K**) Endogenous POLRMT was immunopurified from scrambled control or *FLT3*-knockdown MOLM14 cells to determine K622 succinylation. K622sc levels were normalized to POLRMT protein (ratio) (**J**). Cellular succinyl-CoA levels were determined and normalized to total protein (**K**). Data represent means ± SD, $n = 3$ independent biological replicates, *t* test. $^{**}P < 0.01$. (**L**, **M**) MOLM14 cells were treated with FLT3-IN-3 for 24 h. Endogenous POLRMT was immunopurified to determine K622 succinylation. K622sc levels were normalized to POLRMT protein (ratio) (**L**). Cellular succinyl-CoA levels were determined and normalized to total protein (**M**). Data represent means ± SD, $n = 3$ independent biological replicates, *t* test. $^{**}P < 0.01$. (**N**) mRNA expression of mitochondrial genes in TCGA AML dataset as indicated was extracted from cBioportal. Gene expression levels in $FLT3^{WT}$ ($n = 116$) and $FLT3^{TKD/ITD}$ ($n = 39$) samples were compared. *t* test. $^{*}P < 0.05$. (**O–R**) MV411 cells were treated with FLT3 inhibitors. Protein expression of indicated genes was determined by western blotting (**O**). Enzyme activities of SDH (**P**) and FH (**Q**) were assayed and normalized to total protein. Endogenous POLG was immunoprecipitated to evaluate lysine succinylation (**R**). Data represent means ± SD, $n = 3$ independent biological replicates, *t* test. $^{**}P < 0.01$; n.s. not significant. (**S**) Scrambled control or shRNAs targeting *FLT3* (#1) and *SUCLG1* (#1) were co-expressed in MV411 cells as indicated. Knockdown efficiency was tested by western blotting. Source data are available online for this figure.

© The Author(s)

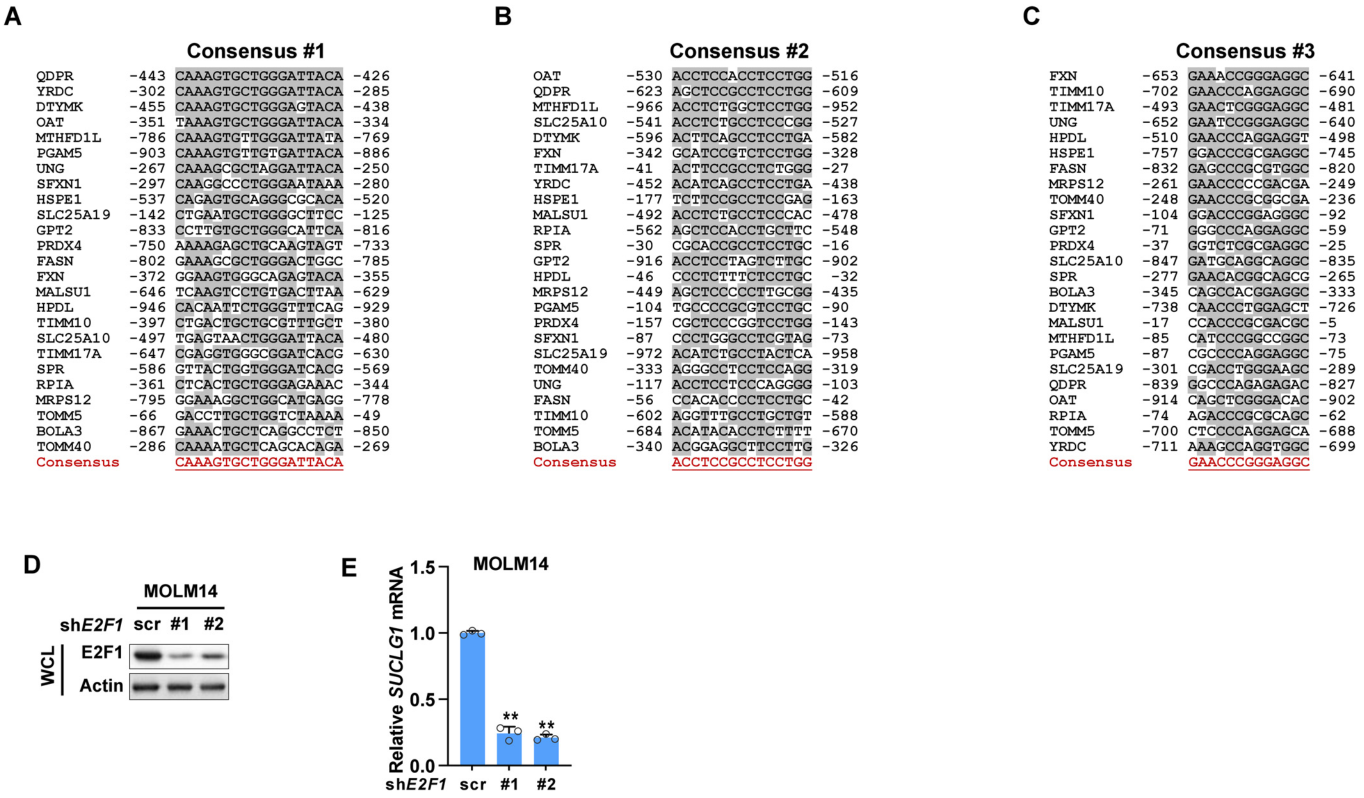

Figure EV5. FLT3 signaling modulates nuclear transcription to enhance mitochondrial respiration.

(**A–C**) Multiple alignments of consensus sequences in FLT3-regulated mitochondrial genes. Numbers indicate the distance (bp) to TSS. (**D**, **E**) Scrambled control or shRNAs targeting *E2F1* were expressed in MOLM14 cells. The knockdown efficiency was validated with western blotting (**D**). Total RNA was extracted to determine the mRNA expression of *SUCLG1* (**E**). Data represent means ± SD, $n = 3$ independent biological replicates, *t* test. $^{**}P < 0.01$. Source data are available online for this figure.

 © The Author(s)

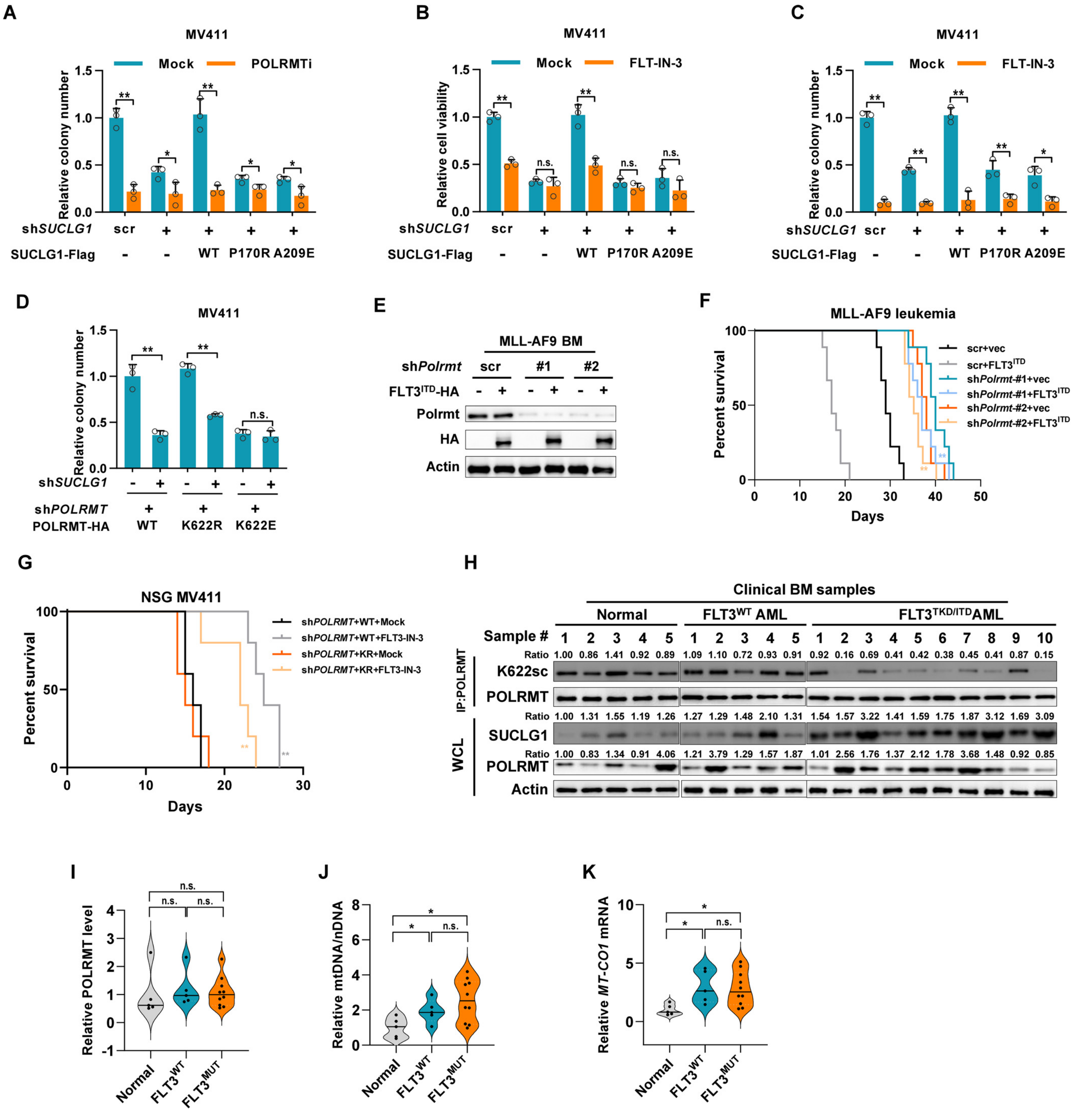

Figure EV6. SUCLG1 suppresses POLRMT succinylation to support leukemic proliferation.

(**A**) *SUCLG1*-knockdown and rescue MV411 cells were treated with POLRMT inhibitor (POLRMTi). Colony formation abilities were assayed. Data represent means ± SD, $n = 3$ independent biological replicates, *t* test. $^{**}P < 0.01$; $^{*}P < 0.05$. (**B**, **C**) *SUCLG1*-knockdown and rescue MV411 cells were treated with FLT3 inhibitor FLT-IN-3. Cell proliferation (**B**) and colony formation (**C**) were assayed. Data represent means ± SD, $n = 3$ independent biological replicates, *t* test. $^{**}P < 0.01$; $^{*}P < 0.05$; n.s. not significant. (**D**) Scrambled control or shRNA against *SUCLG1* (#1) were stably expressed in *POLRMT*-knockdown and rescue MV411 cells. Colony formation was determined. Data represent means ± SD, $n = 3$ independent biological replicates, *t* test. $^{**}P < 0.01$; n.s. not significant. (**E**, **F**) MLL-AF9 bone marrow cells were transduced with vector control or HA-tagged $FLT3^{ITD}$. Scrambled control or shRNAs targeting *Polrmt* was stably expressed. Protein expression was determined by western blotting (**E**). Animal survival was assayed after bone marrow transplantation (**F**). $n = 9$ mice, log-rank (Mantel–Cox) test. $^{**}P < 0.01$. (**G**) *POLRMT*-knockdown and rescue MV411 cells were transplanted into sublethally irradiated NSG mice. Mice were treated with or without FLT3-IN-3, animal survival was determined. $n = 5$ mice, log-rank (Mantel–Cox) test. $^{**}P < 0.01$. (**H**–**K**) Human clinical AML bone marrow samples were collected. K622 succinylation of immunopurified POLRMT was determined by western blotting (**H**). Protein expression of POLRMT (**I**), mtDNA abundance (**J**), and *MT-CO1* mRNA expression (**K**) were determined. $n = 5$–10, *t* test. $^{*}P < 0.05$; n.s. not significant. Source data are available online for this figure.
